# L-asparaginase from the novel *Fusarium falciforme* AUMC 16563: extraction, purification, characterization, and cytotoxic effects on PC-3, HePG-2, HCT-116, and MCF-7 cell lines

**DOI:** 10.1186/s12866-025-03833-8

**Published:** 2025-03-17

**Authors:** Abdullah Abobakr Saleh, Hamdy M. El-Aref, Azza M. Ezzeldin, Rania M. Ewida, Osama A. M. Al-Bedak

**Affiliations:** 1 Molecular Biology Researches & Studies Institute, Assiut University, Asyut, Egypt; 2 South Egypt Cancer Institute, Department of Clinical Pathology and Hematological Malignancies, Assiut University, Asyut, 71511 Egypt; 3 Department of Genetics, Faculty of Agriculture, Assiut University, Assiut, 71511 Egypt; 4 Clinical Pathology Department, Faculty of Medicine, Assiut University, Asyut, 71511 Egypt; 5 Food Hygiene, Safety and Technology Department, Faculty of Veterinary Medicine, New Valley University, El-Kharga, 72511 Egypt; 6 Assiut University Mycological Centre, Assiut, 71511 Egypt; 7 ERU Science & Innovation Center of Excellence, Egyptian Russian University, Badr City, 11829 Egypt

**Keywords:** L-asparaginase, *Fusarium falciforme*, Optimization, Submerged fermentation, Cancer therapy, Cell lines, Gene expression, Apoptosis

## Abstract

**Background:**

L-asparaginase has been a widely employed as antitumor enzyme for the treatment of acute lymphoblastic leukemia for almost three decades. The enzyme takes advantage of the inability of tumor cells to synthesize the L-asparagine and is killed by L‐asparagine deprivation. Despite the availability of bacterial sources for L-asparaginase, there is a growing interest in identifying new microbial sources with improved therapeutic properties. Therefore, this study aims to investigate the production of L-asparaginase from a fungal source, to explore its potential as a novel alternative enzyme for cancer treatment.

**Results:**

*Fusarium falciforme* AUMC 16563 was used to produce L-asparaginase (123.42 U/mL) after 5 days, 0.2% glucose and 1.0% asparagine; were used at 25 **˚**C and pH 8.0. Employing two columns of chromatography (DEAE-cellulose and Sephacryl S 200 HR), the enzyme was purified 14.26-fold, reaching a maximum activity of 5109.4 U/mg. SDS-PAGE revealed a 46.06 kDa asparaginase. The *K*_m_ and *V*_max_ values for pure asparaginase using asparagine was 5.77 × 10^− 2^ mM and 128.22 µmol/min. Additionally, *Fusarium falciforme* AUMC 16563’ pure asparaginase demonstrated anticancer activity against PC-3 (a prostate cell line) with an IC_50_ of 78.6 µg/mL, HePG-2 (a human hepatocellular carcinoma cell line) with an IC_50_ of 69.6 µg/mL, HCT-116 (a colon cell line) with an IC_50_ of 51.5 µg/mL and MCF-7 (a breast cancer cell line) with an IC_50_ of 32.8 µg/mL. The expression levels of proapoptotic genes (BAX and p53) were significantly greater in the breast cancer cell lines treated with asparaginase than in the negative control breast cancer cell lines.The degree of DNA fragmentation in MCF-7 cells treated with *Fusarium falciforme* 16563’ pure asparaginase was 27.2 ± 0.69%, and that in MCF-7 cells treated with the drug Doxorubicin 24.1 ± 0.86% was significantly greater than that in the corresponding negative control cells 9.1 ± 1.01%. Finally, the biochemical profiles revealed no impact on the liver or the kidneys. These results suggested that asparaginase had relatively little effect on liver function. All hematological parameters were within normal range during the experiment.

**Conclusions:**

The results of the present study revealed a potent L-ASNase from endophytic *F. falciforme* isolated from *Trifolium alexandrinum*, which performs well under a variety of environmental circumstances and can be used in a number of commercial applications.

**Supplementary Information:**

The online version contains supplementary material available at 10.1186/s12866-025-03833-8.

## Introduction

L-asparaginase (L-asparagine amidohydrolase, E.C. 3.5.1.1) is an amidase that promotes the hydrolysis of L-asparagine, releasing ammonia and L-aspartic acid [1, 2]. Due to their inability to produce asparagine synthetase, neoplastic cells must rely on extracellular L-asparagine for protein synthesis. According to Amena et al. [3]. and Fontes et al. [4]., L-asparaginase has the ability to degrade L-asparagine in plasma. Because of this, the concentration of L-asparagine declines dramatically, which stops cancer cells from multiplying and stops protein synthesis. The fact that asparagine synthetase allows cells to make their own essential amino acids implies that normal animal cells can survive without L-asparagine [5]. By reducing L-asparagine levels and targeting neoplastic cells dependent on this amino acid, L-asparaginase acts as an anticancer drug when administered intravenously [6].

L-asparaginase is a chemotherapeutic drug commonly used for the treatment of several lymphoproliferative diseases and lymphomas, particularly acute lymphoblastic leukemia (ALL). For almost three decades, it has played a vital role in combination chemotherapy protocols for the therapy of pediatric acute lymphoblastic leukemia [7].

This enzyme is essential in the pharmaceutical and biomarker industries [8] and shows promising therapeutic effects when combined with other drugs for the treatment of cancers such as melanosarcoma, reticulohistocytosis, lymphocytic leukemia, Hodgkin’s lymphoma, chronic lymphosarcoma, acute myelomonocytic leukemia, acute myelocytic leukemia, acute lymphoblastic leukemia [9–14]. Asparagine is used as a biomarker to assess levels of the carcinogenic chemical acrylamide during chemotherapy and is also used in the food industry as an acrylamide neutralizer [15, 16].

The ease of growing microbes and extracting and purifying their enzymes makes them a promising source of L-asparaginase, which can be produced on a large scale [17]. Isolating microbial strains that synthesize this crucial enzyme has been the focus of numerous studies. These strains include *Pseudomonas fluorescens* [18], *Serratia marcescens* [19], *Escherichia coli* [20], *Erwinia carotovora* [21], *Proteus vulgaris* [22], *Saccharomyces cerevisiae*, *Streptomyces venezuelae*, and several fungal genera like *Aspergillus*, *Penicillium*, and *Fusarium* [23].

The fact that this enzyme originates from bacteria means that it can set off allergic reactions [24]. In 2007, the safety of fungal L-asparaginases was determined by the Joint FAO/WHO Expert Committee on Food Additives (JECFA). The current production levels of L-asparaginase did not satisfy the demand. Therefore, new approaches to increasing yield, perhaps aided by statistical methods, must be developed [25]. Hypersensitivity, antigenicity, limited half-life, transient blood clearance, and L-glutaminase-dependent neurotoxicity are some of the challenges that have been reported with L-asparaginases derived from *E*. *coli* and *Erwinia chrysanthemi*. These issues highlight the need for ongoing research to find better alternatives [26–28].

Given the problems associated with commercially available L-asparaginases, scientists are currently looking for alternative sources of the enzyme. Hence, the present investigation intended to isolate L-asparaginase from the endophytic wild-type *Fusarium falciforme* AUMC 16563 strain and characterize it. In vitro testing using PC-3, HePG-2, HCT-116, and MCF-7 cell lines allowed for the evaluation of the isolated enzyme’s cytotoxicity. To round out the optimal results from the cell lines examined, an analysis of the Bcl-2, BAX, and p53 genes were performed, which was supplemented by an in vivo evaluation in an animal model.

## Materials and methods

### Assay of L-asparaginase

Nesslerization method [29] was used to conduct the enzyme assay. The ammonia that was released was quantified by measuring the absorbance at 480 nm, Using ammonium sulfate as a reference standard. Under standard assay circumstances, one unit of L-asparaginase is the amount of enzyme that releases 1 µmol of ammonia.

### Fungal strain

A potential *Fusarium* isolate was found to possess remarkable asparaginase activity during the screening of L-asparaginase activities in several endophytic fungi. In order to optimize the fermentation parameters and conduct molecular identification, this isolate was chosen. The fungus was maintained in the culture collection of the Assiut University Mycological Centre as AUMC 16563.

### Medium for fermentation

Sucrose-free Czapek’s broth medium supplemented with L-asparagine was used as fermentation medium. The medium contained (g/L): Asparagine, 10; sodium nitrate, 2.0; dipotassium hydrogen orthophosphate, 1; magnesium sulphate, 0.5; potassium chloride, 0.5; zinc sulphate, 0.01; copper sulphate, 0.005 [30].

### Optimization of fermentation parameters

At different pH values (4–10), nitrogen sources (ammonium chloride, ammonium sulphate, sodium nitrate, sodium nitrite, urea, peptone, yeast extract, and beef extract) each at 0.2%, incubation temperatures (25, 30, 35, 40, 45, and 50 °C), and fermentation times (1–7 days), the optimization of L-asparaginase production by the *Fusarium* isolate AUMC 16563 was investigated using one factor at a time (OFAT). The enzyme assay was conducted using the previously mentioned protocol, and the most appropriate parameters for enzyme synthesis were selected.

**Molecular identification of the*Fusarium*isolate**.

DNA of the *Fusarium* isolate AUMC 16563 was isolated following the method outlined by Moubasher et al. [31]., PCR reaction was performed using SolGent EF-Taq [32]. ITS1 and ITS4 universal primers were used for amplification of the ITS region [33]. The contiguous sequence of the *Fusarium* isolate AUMC 16563 utilized in this study was produced using the DNASTAR software (version 5.05). The current analysis utilized eighteen species: one of the *Fusarium* isolate AUMC 16563 in this investigation, sixteen of the closely related species were retrieved from the GenBank database, and one sequencing of *Fusarium equiseti* NRRL 26419 served as an outgroup. All sequences were aligned using MAFFT [34] with the default settings. BMGE was employed to optimize alignment gaps and parsimony-uninformative characters [35]. MEGA X (version 10.2.6) was utilized to perform maximum-likelihood (ML) and maximum-parsimony (MP) phylogenetic analyses [36]. The durability of the most frugal trees was evaluated using 1000 bootstrap replications [37]. The optimal nucleotide substitution model for the ML experiments was determined using the Akaike information criterion (AIC) as implemented in Model test 3.7 [38]. MEGA X (version 10.2.6) was utilized to visualize the phylogenetic tree, which was subsequently edited and saved in TIFF format.

### Purification of L-asparaginase

#### Precipitation and dialysis of L-asparaginase

Following incubation, the cell-free supernatant was obtained using centrifugation at 10,000 rpm for 10 min at 4 °C. Absolute ethyl alcohol (-25 °C) was utilized to isolate the enzyme at 4 °C. The extracted protein was solubilized in Tris buffer (pH 8.0), subjected to dialysis twice for two hours at ambient temperature (cutoff: 12–14 kDa), and subsequently refrigerated overnight at 4 °C to exclude salts and other small molecules [39].

### DEAE-cellulose ion exchange column

The diethylaminoethyl cellulose (DEAE-cellulose) anion exchanger that was activated with 0.5 M NaOH for 60 min, was packed in a glass column (50 cm × 2.4 cm; bed volume 200 cm³). A 10.0 mL sample was introduced to the column subsequent to its equilibration with phosphate buffer (100 mM, pH 8.0). The enzyme was eluted using a 100 mM phosphate buffer (pH 8.0) at NaCl concentrations of 0, 0.1, 0.25, 0.5, 1.0, and 1.5 M. 5mL fractions were collected at a flow rate of 0.25/min and assayed for activity. The aforementioned method was employed to quantify asparaginase activity. The most active fractions were pooled, concentrated, and preserved for subsequent purification.

### Sephacryl S 200 HR gel filtration column

Sephacryl S 200 HR gel was packed in a glass column measuring 50 cm × 2.4 cm, with a bed capacity of 200 cm³. The protein was eluted using a phosphate buffer (100 mM, pH 8.0) following the loading of the concentrated enzyme sample onto the column. The asparaginase activity was assessed using the previously described method, 5 ml fractions were collected at a flow rate of 0.25/min and assayed for activity. The most active portions were aggregated, condensed, and freeze-dried.

### SDS˗PAGE

A 0.1 g sample of asparaginase was dissolved in 100 µL of 20 mM Tris/HCl, pH 7.4 containing 4.0% sodium dodecyl sulphate (SDS), 20% glycerol, 10% 2-mercaptoethanol, and 0.0025% bromophenol blue. A 12% SDS polyacrylamide gel was prepared, subjected to heating at 100 °C for five minutes, and electrophoresed at 100 mA and 150 V for 45 min. Coomassie Brilliant Blue R-250 was used for protein staining. Quantity One software (Version 4.6.2) was employed to capture images of the gel [40].

### Effect of pH and temperature on the activity of pure asparaginase

A range of pH values from 3.0 to 11.0 were tested on pure asparaginase at temperatures of 21, 24, 27, 30, 33, 36, 39, 42, and 45 °C. Citrate buffer (pH 3–6), Phosphate buffer (pH 7–8), and Glycine-NaOH buffer (pH 9–11) were used for pH adjustment. Each buffer was prepared at a concentration of 0.1 M. At the specified pH levels, 0.01 g of enzyme powder and 0.01 g of asparagine were added to 1.0 mL of buffer solution to initiate the reaction. The reaction was stopped by adding 2.0 mL of TCA after 30 min, and the asparaginase activity was quantified as aforementioned.

### Determination of metal ion effect on pure asparaginase

For testing the effect of metal ions on activity of the pure asparaginase, a solution containing 5 mM of the following metal ions was prepared: NaCl, KCl, CaCl_2_, CoCl_2_, NiSO_4_, CuSO_4_, FeSO_4_, MgSO_4_, MnSO_4_, and ZnSO_4_. The metal ions that were present were as follows: Na^+^, K^+^, Ca^2+^, Co^2+^, Ni^2+^, Cu^2+^, Fe^2+^, Mg^2+^, Mn^2+^, and Zn^2+^. Additionally, ethylenediaminetetraacetic acid (EDTA) at 5 mM were used to investigate an enzyme inhibitor. The asparaginase activity under standard reaction conditions, and EDTA-free, was evaluated to determine what 100% activity means. The experiment was repeated three times.

### Determination of *K*_m_ and *V*_max_

Using a Line weaver-Burk plot [41] in accordance with Eq. (1), the enzyme activity was measured at concentrations ranging from (2.0–20.0 mM) of L-asparagine, L-glutamine, aspartic acid, and glutamic acid as substrates to find the Michaelis-Menten constant (*K*_m_) and maximum reaction velocity (*V*_max_) values of the pure asparaginase.

1 $$\:\frac{1}{\text{v}}=\frac{1}{{V}_{max}}+\frac{{\text{K}}_{\text{m}}}{{V}_{max}}\:\times\:\:\frac{1}{\text{S}}$$

### Cytotoxic effects on human cell lines

#### Source of cell lines

The cell lines used in this study (HePG-2, HCT-116, PC-3, and MCF-7) were obtained from the Cell Culture Laboratory, National Research Centre, El-Tahrir St., Dokki, Giza 12622, Egypt.

### MTT assay

Bioassay-Cell Culture Laboratory at the National Research Centre in Giza, Egypt, conducted the in vitro bioassays of the pure L-asparaginase on human tumor cell lines HePG-2, HCT-116, PC3, and MCF-7. The cell lines were suspended in RPMI 1640 medium supplemented with a 1.0% antibiotic/antimycotic combination (10,000 U/mL penicillin G potassium, 10,000 µg/mL streptomycin sulphate, and 25 µg/mL amphotericin B) and 1.0% L-glutamine, and maintained at 37 °C in a 5.0% CO_2_ environment [42]. Cells were cultured in batches for 10 days in a water-jacketed carbon dioxide incubator (Sheldon, TC2323, Cornelius, OR, USA) before being seeded at a concentration of 10 × 10^3^ cells/well in new complete growth medium. This was conducted in a 96-well microtiter plate at 37 °C for 24 h under 5% CO_2_. To achieve final concentrations of 100, 50, 25, 12.5, 6.25, 3.125, 0.78, and 1.56 µg/mL, the medium was aspirated, fresh serum-free medium was added, and the cells were cultured either in isolation (negative control) or with different sample concentrations. After 48 h of incubation, the medium was aspirated, and 40 µL of MTT salt (2.5 µg/mL) was added to each well. The wells were subsequently incubated for four additional hours at 37 °C in a 5% CO_2_ environment. A 200 µL of 10% sodium dodecyl sulphate (SDS) in deionized water were added to each well and incubated overnight at 37 °C to terminate the reaction and dissolve the formed crystals. Doxorubicin at a concentration of 100 µg/mL served as the positive control [43, 44]. A microplate multiwell reader (Bio-Rad Laboratories Inc., model 3350, Hercules, California, USA) was employed to measure absorbance at 595 nm, facilitating the determination of the IC_50_ and the optimal concentration of the tested cell lines for subsequent molecular experiments. The percentage change in viability was determined using Eq. (2).

% Cell viability = [($$\:\frac{\mathbf{R}\mathbf{e}\mathbf{a}\mathbf{d}\mathbf{i}\mathbf{n}\mathbf{g}\:\mathbf{o}\mathbf{f}\:\mathbf{s}\mathbf{a}\mathbf{m}\mathbf{p}\mathbf{l}\mathbf{e}\:}{\mathbf{R}\mathbf{e}\mathbf{a}\mathbf{d}\mathbf{i}\mathbf{n}\mathbf{g}\:\mathbf{o}\mathbf{f}\:\mathbf{n}\mathbf{e}\mathbf{g}\mathbf{a}\mathbf{t}\mathbf{i}\mathbf{v}\mathbf{e}\:\mathbf{c}\mathbf{o}\mathbf{n}\mathbf{t}\mathbf{r}\mathbf{o}\mathbf{l}})\:$$**–** 1] × 100 (2).

### DNA fragmentation assay

#### DNA gel electrophoresis laddering assay

DNA fragmentation assay was conducted following the instructions provided by Yawata et al. [45] for the chosen cell lines. The cell lines were homogenized in one mL of RPMI 1640 medium and subsequently exposed to various compounds, including L-asparaginase from *F. falciforme* AUMC 16563 and the drug doxorubicin. The cells were subsequently centrifuged at 800 rpm for a duration of 10 min. The cells were subsequently harvested and rinsed with Dulbecco’s phosphate-buffered saline. MCF-7 cells were lysed on ice for 30 min using a lysis mixture composed of 10 mM Tris base (pH 7.4), 150 mM NaCl, 5 mM EDTA, and 0.5% Triton X-100. The lysates were centrifuged for 20 min at 10,000 rpm. DNA fragmentation was extracted from the supernatant utilizing an equal volume of a neutral phenol, chloroform, and isoamyl alcohol mixture in a ratio of 25:24:1. The samples underwent electrophoretic analysis on 2.0% agarose gel containing 0.1 µg/mL ethidium bromide.

### Diphenylamine reaction procedure

Asparaginase from *Fusarium falciforme* AUMC 16563 and the chemotherapy drug doxorubicin were added to the cells as soon as they were taken from the culture. Cancer cells were spun for 20 min at 4 ºC at 10,000 rpm after being lysed in 0.5 mL of lysis buffer that contained 10 mM Tris-HCl (pH 8.0), 1.0 mM EDTA, and 0.2% Triton X-100. To resuspend the pellet, 0.5 mL of lysis buffer was added. The mixture was incubated at 4 °C for 24 h after adding 0.5 mL of 25% Trichloroacetic acid (TCA) to the pellets and supernatants. Following a 20-minute centrifugation at 10,000 rpm and 4 °C, the cell pellets were mixed with 80 mL of 5.0% TCA and left to incubate at 83 °C for another 20 min. Following this, each cell sample was treated with 160 mL of a diphenylamine (DPA) solution, which included 150 mg of DPA in 10 mL of glacial acetic acid, 150 mL of sulphuric acid, and 50 mL of acetaldehyde (16 mg/mL). According to Gibb et al. [46], the samples were subsequently left to incubate at room temperature for 24 h. Equation (3) was used to determine the proportion of fragmented DNA based on the absorbance observed at 600 nm.

% Fragmented DNA = $$\:\frac{\text{O}\text{D}\:\left(\text{S}\right)}{\text{O}\text{D}\:\left(\text{S}\right)+\:\text{O}\text{D}\:\left(\text{P}\right)}$$× 100 (3)

where: OD = optical density; S = supernatants; and P = pellets.

### Gene expression analysis

Following the instructions provided by the manufacturer, total RNA was extracted from the cell lines that were evaluated using an RNeasy Mini Kit (Qiagen, Hilden, Germany) in conjunction with the Qiagen DNaseI digestion step. One unit of RQ1 RNAse-free DNAse (Invitrogen, Germany) was used to digest the DNA residues, and then the isolated total RNA was resuspended in water treated with diethyl pyrocarbonate (DEPC). According to Linjawi et al. (2017) [47], the quantity was measured at 260 nm. A 20 µL of full poly (A) + RNA isolated from the cell lines were reverse transcribed using a RevertAidTM First Strand cDNA Synthesis Kit (manufactured by Fermentas in Germany). After a denaturation step of 5 min at 99 °C, the RT reaction was conducted for 10 min at 25 °C, followed by one hour at 42 °C. The number of cDNA copies in the cell lines was determined using an Applied Biosystems StepOneTM Real-Time PCR System (Thermo Fisher Scientific, Waltham, MA USA). According to Brito, et al. [48] and Khalil, et al. [49], the primer sequences that were created for the Bcl-2, BAX, and p53 genes, which were cancer-associated and related to the cell lines were listed in Table 1. According to Ramadan, et al. [50] and Refaie, et al. [51], the relative quantification of the target in comparison to the reference was determined using the 2 − ΔΔCT method.

**Table 1:** Sequences of primers used for qRT-PCR of the PC-3, HePG-2, HCT-116, and MCF-7 cell lines

Gene	GenBank (accession no.)	Primer sequence
*Bcl-2*	M14745.1	F: cct cgc tgc aca aat act cc R: tgg aga gaa tgt tgg cgt ct
*BAX*	XM_054373112.1	F: ctg tat gtg gga ctg gtg gt R: gga aat gag ggg tgg aag ga
*p53*	X60020.1	F: tgg cca. tct aca agc agt ca. R: ggt aca gtc aga gcc aac ct
*GAPDH*	AK026525.1	F: cac atc gct cag aca cca tg R: tga cgg tgc cat gga att tg

BCL-2: B-cell lymphoma-2 gene; BAX: Bcl-2-associated X protein encoding gene; p53: tumor suppressor gene; GAPDH: housekeeping gene

### In vivo cytotoxicity of L-asparaginase in mice

Prior to the initiation of all experiments, all mice were transferred into a dedicated experimental room where they were maintained in sterile micro-isolator cages, and housed on ventilated racks, with up to 5 mice in a cage with corncob bedding. The mice had access to food and water ad libitum. Approximately 40 g Swiss albino mice, six to eight weeks old, were utilized in the experiment. This experiment would not have been possible without the kind donation of mice from the Veterinary Teaching Hospital at Assiut University in the Assiut Governorate in Egypt. There were two sets of ten mice in the total. *F. falciforme* AUMC 16563’ L-asparaginase was injected into the second group at a dosage of 500 IU/kg, while the first group received 1X phosphate-buffered saline twice weekly as a control. The experiment lasted for seven weeks. Fifteen, thirty and forty- five days following the last injection, at the time of sacrifice, mice were anesthetized with 2% isoflurane, and blood was collected via cardiac puncture. Plasma was frozen at -40 °C until assayed. Regarding the euthanasia procedure for the animals, all animals were anesthetized prior to euthanasia. We kept the serum and plasma at -40 °C after extraction [52, 53], and others have measured the full blood count in addition to transaminases, albumin, total protein, urea, creatinine, and alkaline phosphatase levels. The toxicity markers were assayed in the Clinical Pathology Department’s Hematology and Clinical Chemistry laboratories at the South Egypt Cancer Institute, Assiut University, Assiut, Egypt. The measurements were taken using universal biochemical keys.

### Statistical analysis

All data were expressed using the mean and standard deviation (SD) of the triplicate preliminary research. The statistical significance was analyzed [54], and variances were considered statistically significant when *p* ≤ 0.05.

## Results

### Molecular identification of the *Fusarium* isolate AUMC 16563

**Fig. 1:**
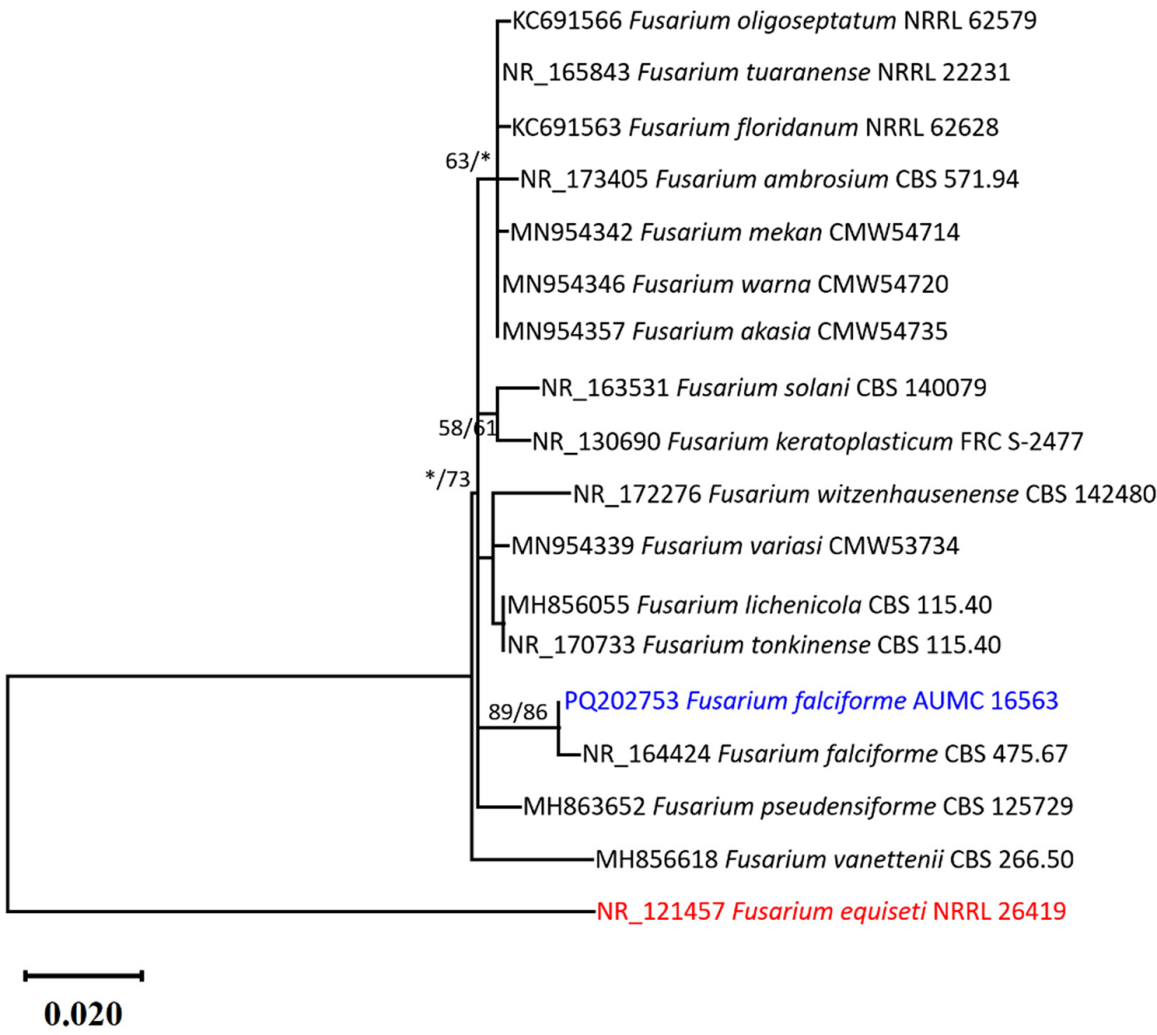
Maximum likelihood phylogenetic tree generated from ML/MP analysis via a heuristic search (1000 replications) of the 18 S sequence of *Fusarium falciforme* AUMC 16563 (in blue) compared with other closely related species to the genus *Fusarium* in GenBank. Bootstrap support values for ML/MP ≥ 50% are indicated near the respective nodes. The tree is rooted to *Fusarium equiseti* NRRL 26419 as an out group (in red)

The entire ITS dataset comprised 18 species. The maximum parsimony dataset consisted of 588 characters with 468 constant characters (no gaps, no N), 74 variable characters which were parsimony-uninformative (15.8% of constant characters), and 5 characters were counted as parsimony informative (1.1% of constant). Kimura 2-parameter using a discrete Gamma distribution (K2 + G) was the perfect model for substitution of nucleotides. The dataset for maximum parsimony yielded five trees, the most parsimonious one with the highest log likelihood of -1446.80, tree length of 118 steps, consistency index of 0.681818, retention index of 0.766667, and composite index of 0.522727 is shown in Fig. 1. The *Fusarium* isolate in this study was placed with the type species *F. falciforme* CBS 475.67 at the same clade endorsing strong supported clade (89% ML/86% MP). As a result, these isolates in the present study are identified here as *F. falciforme* (Fig. 1).

### Optimization of L-asparaginase production parameters

**Fig. 2:**
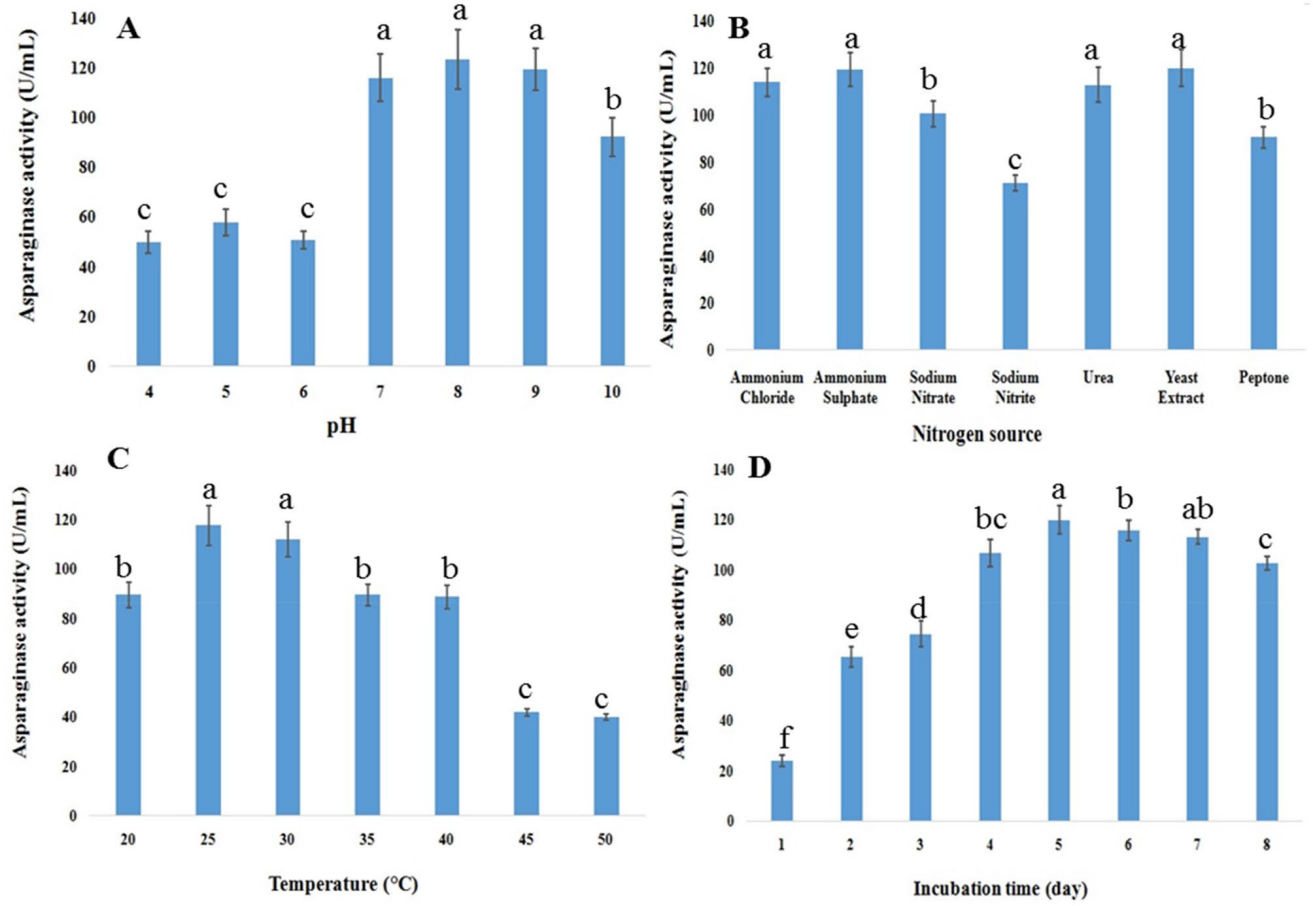
Effect of fermentation parameters (**A**) medium’s pH (**B**) nitrogen supply (**C**) incubation temperature (**D**) incubation time, on the activity of asparaginase produced by *F. falciforme* AUMC 16563 in SmF (Means with different values between treatments in the same column are significantly different at *p* < 0.05)

**Fig. 3:**
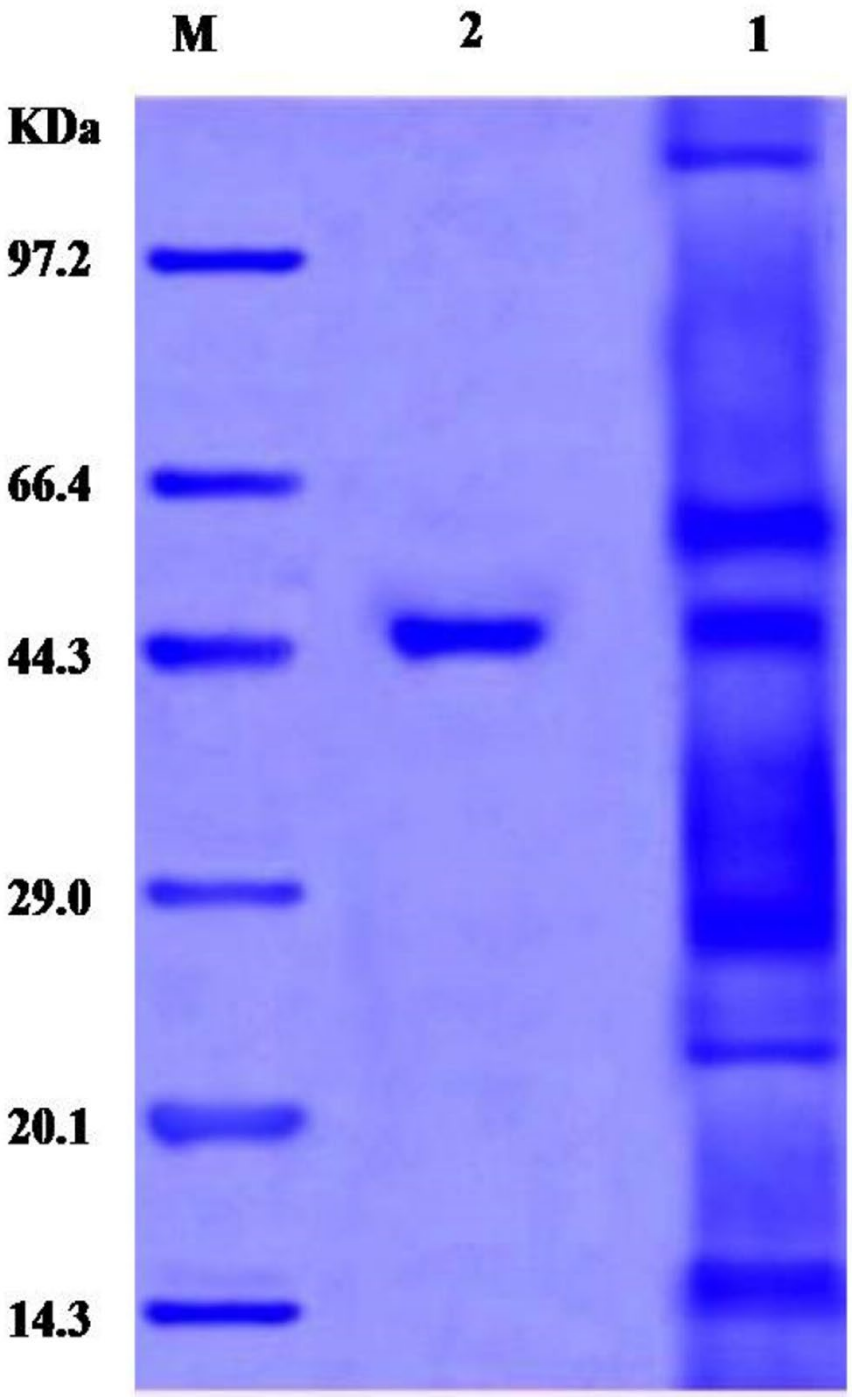
SDS-PAGE of asparaginase produced by *F. falciforme* AUMC 16563. M: prestained marker. Lane 1: crude enzyme. Lane 2: pure asparaginase

Following the elimination of all nitrogen sources from the fermentation medium, with the exception of asparagine, this study determined that *F. falciforme* AUMC 16563 exhibited peak asparaginase activity at pH 8.0, measuring 123.42 ± 12 U/mL (Fig. 2A). The introduction of a nitrogen source to the fermentation medium resulted in a reduction of asparaginase activity. Yeast extract emerged as the most effective nitrogen source, resulting in an asparaginase activity of 119.92 ± 7.2 U/mL (Fig. 2B). Figure 3 indicates that asparagine served as the exclusive nitrogen source, resulting in a significant increase (*p* < 0.05) in asparaginase activity to 120.19 ± 5.6 U/mL at 25 ºC after 5 days of incubation (Fig. 2C–D).

### Purification of asparaginase

*F. falciforme* synthesized asparaginase after five days of cultivation at pH 8.0 and 25 °C, utilizing asparagine as the sole nitrogen source. The DEAE-cellulose gel yielded 74 pooled fractions (Figure S1), exhibiting the highest peaks of active asparaginase and protein. Following the collection of the highest-activity fractions (18–90) from the DEAE-cellulose column, further purification was conducted using a Sephacryl S-200 HR column. The asparaginase components from *F. falciforme* were purified using Sephacryl S-200 HR (Fractions no. 17–70), resulting in two significant broad peaks of asparaginase and protein activity (Figure S2). After two processing cycles, the specific activity of the purified asparaginase increased by 14.26-fold, yielding a protein concentration of 2.38% and a specific activity of 5109.4 U/mg (Table 2).

**Table 2:** Purification profile of asparaginase produced by *F. falciforme* AUMC 16563

Purification steps	Volume (mL)	Total activity (U)	Total protein (mg)	Specific activity (U/mg)	Yield %	Fold
Fermentation medium	1330	129,409	361.36	358.11	100	1
Ethyl alcohol	105	37,138.5	41.5	894.9	11.48	2.49
DEAE-cellulose	60	40,104	21.3	1882.8	5.9	5.26
Sephacryl S 200 HR	37	43,941.2	8.6	5109.4	2.38	14.26

### SDS-PAGE

Sodium dodecyl sulphate polyacrylamide gel electrophoresis, the examination revealed that the asparaginase produced by *F. falciforme* AUMC 16563 was homogeneous and entirely pure. The molecular weight was determined to be 46.06 kDa (Fig. 3).

### Effects of pH and temperature on the activity of pure asparaginase

**Fig. 4:**
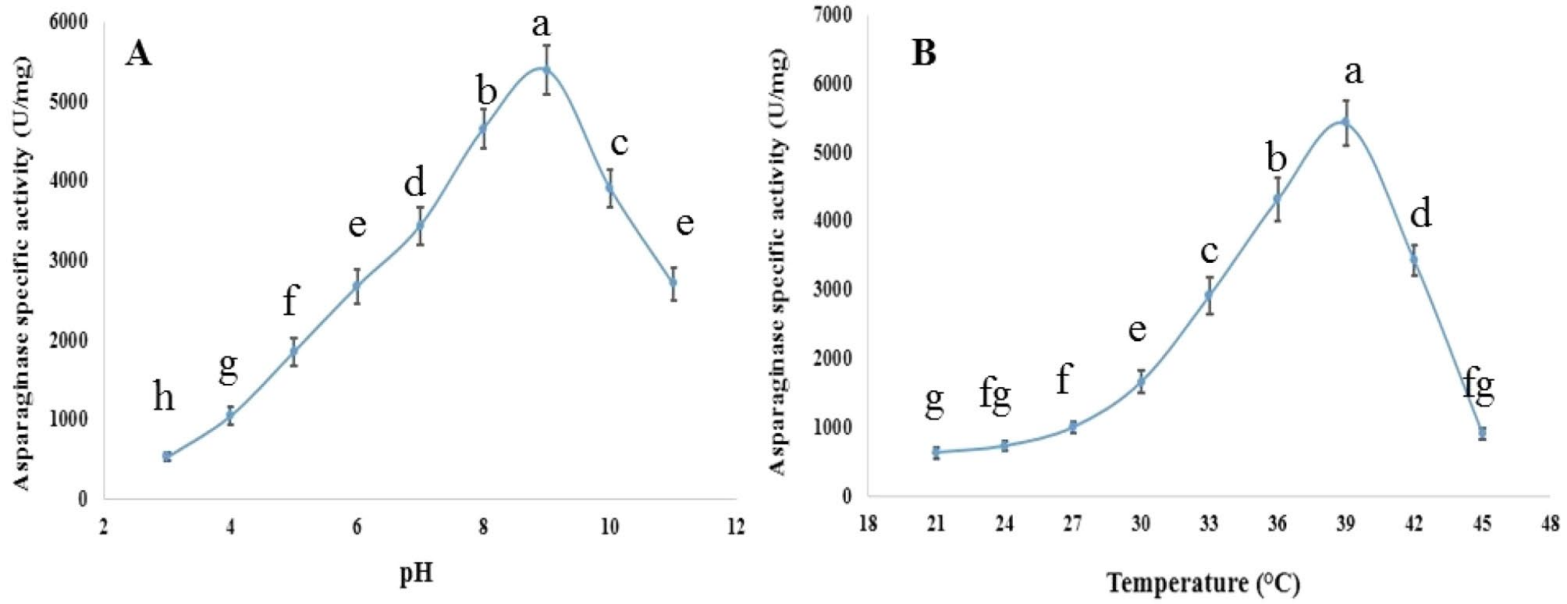
Effects of (**A**) pH and (**B**) temperature on the activity of pure asparaginase produced by *F. falciforme* AUMC 16563 (Means with different values between treatments in the same column are significantly different at *p* < 0.05)

The present study revealed that pure asparaginase exhibited increased activity with the pH increase from 3 to 9, then it declined. pH 9.0 was identified as the optimal condition, achieving a peak activity of 5383.37 ± 310 U/mg at 39 °C (Fig. 4).

### Determination of metal ion effect on pure asparaginase

It could be observed that, there was a significant reduction in the enzyme activity in the presence of Mg^2+^, Co^2+^, Mn^2+^, Cu^2+^, Zn^2+^, Ba^2+^, and Cd^+^ by 30, 45, 38, 83.4, 51.4, 61.5, and 85.5% respectively. K^+^ exerted highly stimulatory effect to occupy the first rank among all tested compounds followed by Na^+^, Ca^2+^, and Fe^2+^ by significant increasing 55, 44.6, 40 and 18.8%, respectively over the control value. The metal chelating agent EDTA didn’t affect L-asparaginase activity (Table 3).

**Table 3:** Effect of 5 mM addition of metal ions and EDTA on the pure asparaginase activity produced by *F. falciforme* AUMC 16563. Residual activity (%) results are expressed as the proportion of the activity in the tested inhibitory conditions, from the asparaginase activity in the control without inhibitors (means with different values between treatments in the same column are significantly different at *p* < 0.05)

Metal ions	Specific activity (U/mg)	Residual activity (%)
Control	5220.23^d^	100±0.0^d^
Na^+^	7548.45^b^	144.6±2.0^b^
K^+^	8091.35^a^	155±1.5^a^
Ca^++^	7308.32^b^	140±1.0^b^
Mg^++^	3654.16^e^	70±0.5^e^
Fe^++^	6201.63^c^	118.8±1.5^c^
Cu^++^	866.56^i^	16.6±0.5^i^
Zn^++^	2537.03^g^	48.6±0.5^g^
Mn^++,^	3236.54^f^	62.0±1.5^ef^
Ba^++^	2009.78^h^	38.5±1.0^h^
Co^++^	2871.13^g^	55±1.5^fg^
Cd^++^	756.93^i^	14.5±0.5^i^
EDTA^++^	5220.23^d^	100±1.0^d^

### Determination of kinetic parameters (***K***_m_ and ***V***_**max**_) and substrate specificity

*K*_m_ and *V*_max_ values were assessed using asparagine, glutamine, aspartic acid, and glutamic acid as substrates at different concentrations (2.0–20.0 mM). The Line Weaver Burk plots demonstrated that L-asparagine was the most suitable substrate for *F. falciforme* AUMC 16563 asparaginase, exhibiting the highest affinity for the enzyme. The *K*_m_ and *V*_max_ values for asparagine, glutamine, aspartic acid, and glutamic acid were found to be 5.77 × 10^− 2^ mM and 128.22 µmol/min, 9.12 mM and 71.66 µmol/min, 10.1 mM and 62.46 µmol/min, and 12.2 mM and 49.51 µmol/min, respectively (Table 4).

**Table 4:** Kinetic parameters of the substrate specificity of L-asparaginase

Substrate	K_m_ (mM)	V_max_ (µmol/min)
Asparagine	5.77 × 10^− 2^	128.22
Glutamine	9.12	71.66
Aspartic acid	10.1	62.46
Glutamic acid	12.2	49.51

### In vitro cytotoxic effects of pure asparaginase on human cell lines

**Fig. 5:**
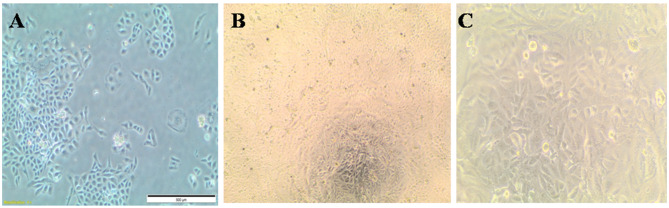
PC-3 cell lines (**A**) untreated cells (negative control) (**B**) PC-3 cells treated with 100 µg/mL doxorubicin (**C**) PC-3 cells treated with *F. falciforme* AUMC 16563’ asparaginase

**Fig. 6:**
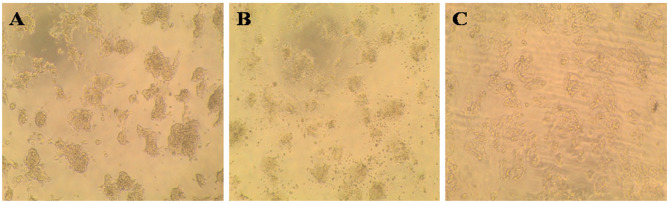
HePG-2 cell lines (**A**) Untreated cells (negative control) (**B**) HePG-2 cells treated with 100 µg/mL doxorubicin and (**C**) HePG-2 cells treated with *Fusarium falciforme* AUMC 16563’ asparaginase

**Fig. 7:**
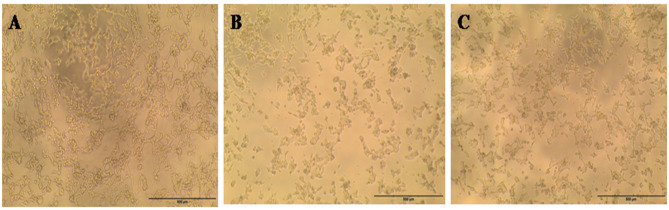
HCT-116 cell lines (**A**) Untreated cells (negative control) (**B**) HCT-116 cells treated with 100 µg/mL doxorubicin, and (**C**) HCT-116 treated with *Fusarium falciforme* AUMC 16563’ asparaginase

**Fig. 8:**
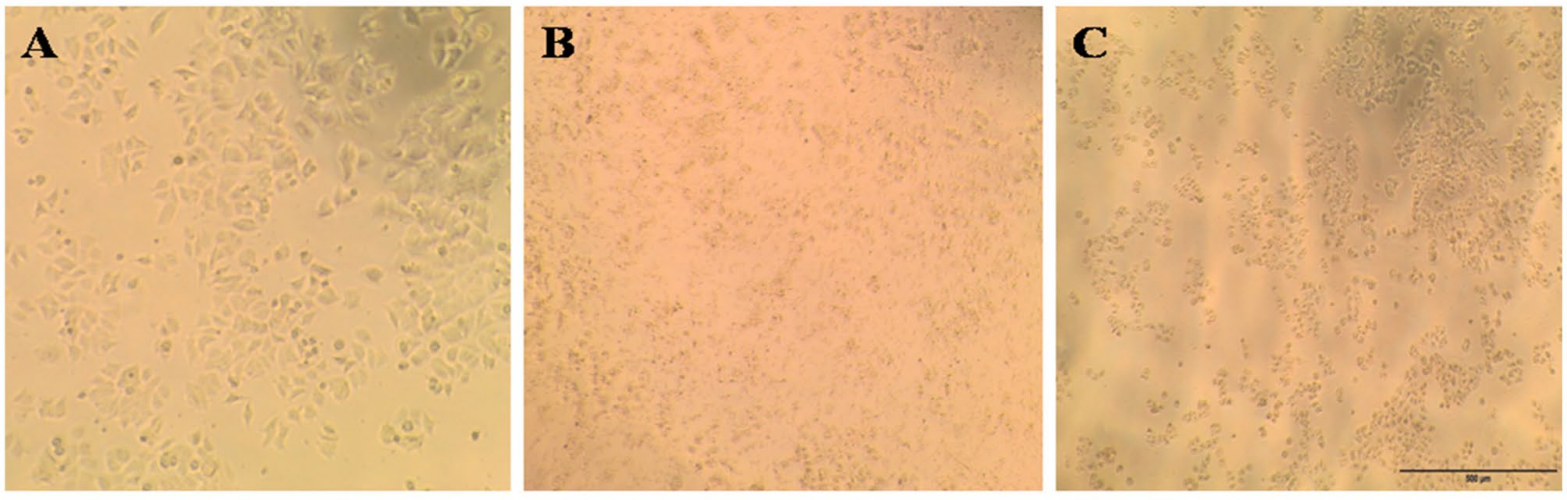
MCF-7 cell lines (**A**) Untreated cells (negative control) (**B**) MCF-7 cells treated with 100 µg/mL doxorubicin, and (**C**) MCF-7 cells treated with *Fusarium falciforme* AUMC 16563’ asparaginase

**Fig. 9:**
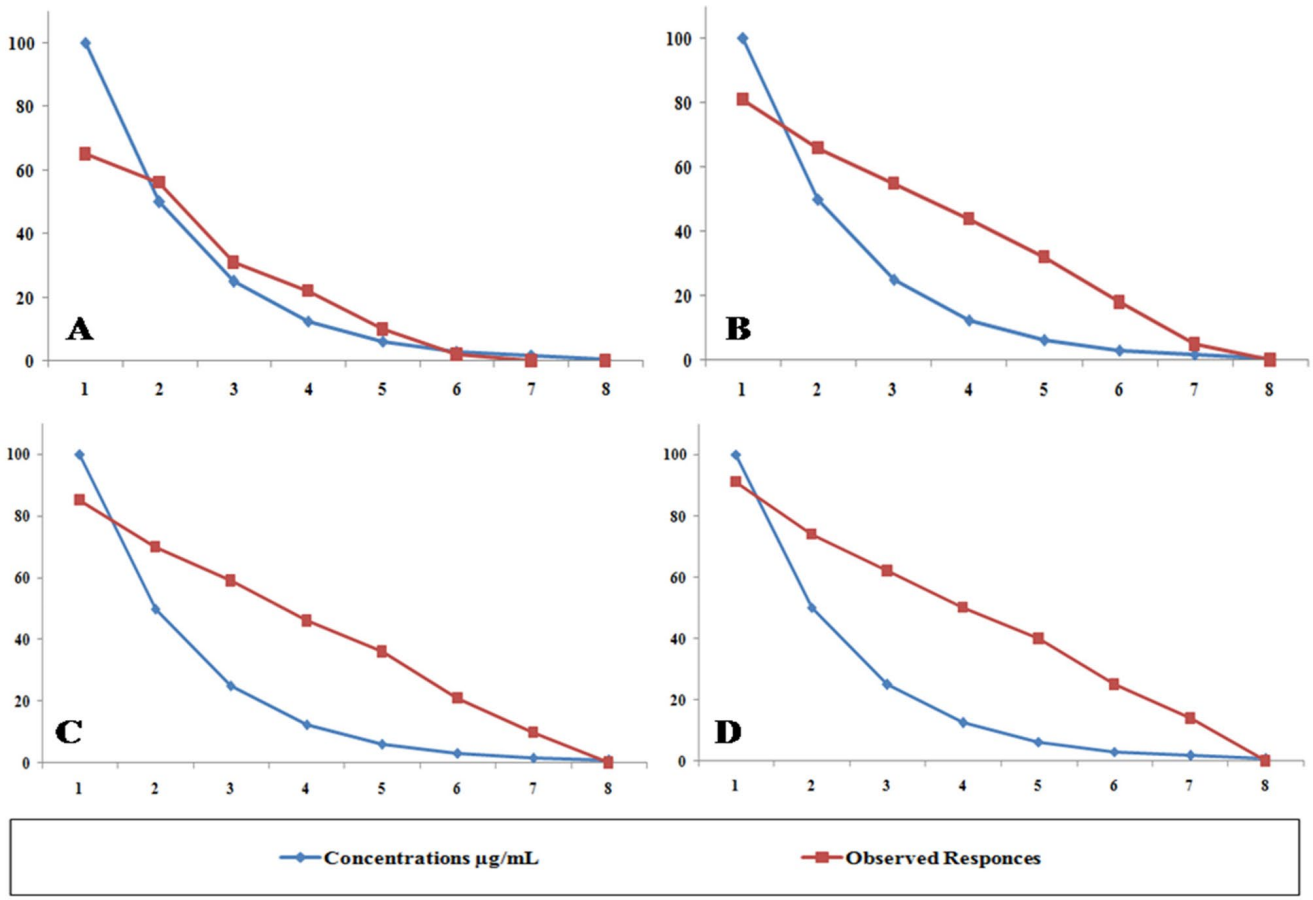
Observed responses of cell lines versus different concentrations (µg/mL) of *F. falciforme* AUMC 16563’ asparaginase (**A**) PC-3 cell line, (**B**) HePG-2 cell line, (**C**) HCT-116 cell line and (**D**) MCF-7 cell line

This study evaluated PC-3, HePG-2, HCT-116, and MCF-7 cell lines. Untreated cell lines served as negative controls (Figs. 5A, 6A, 7A, and 8A); PC-3, HePG-2, HCT-116, and MCF-7 cells exposed to 100 µg/mL doxorubicin acted as positive controls (Figs. 5B, 6B, 7B, and 8B); and PC-3, HePG-2, HCT-116, and MCF-7 cells treated with the IC50 of *F. falciforme*’ asparaginase were also evaluated (Figs. 5C, 6C, 7C, and 8C). The current results showed that as the asparaginase concentration increased, the cell viability of the four cell lines (MCF-7, PC-3, HePG-2, and HCT-116) decreased, suggesting that the cell lines used had distinct sensitivities (Figs. 9A–D). Cytotoxicity in PC-3 cells was significantly induced, yielding an IC50 of 78.6 µg/mL. Cytotoxicity was observed in HePG-2 cells, yielding an IC50 of 69.6 µg/mL. In HCT-116 cells, cytotoxicity resulted in an IC50 of 51.5 µg/mL, while MCF-7 cells exhibited an IC50 of 32.8 µg/mL. These findings indicate the most significant effect of the tested enzyme on the cell lines and were utilized in subsequent investigations to confirm its apoptotic effect.

### Gene expression

**Fig. 10:**
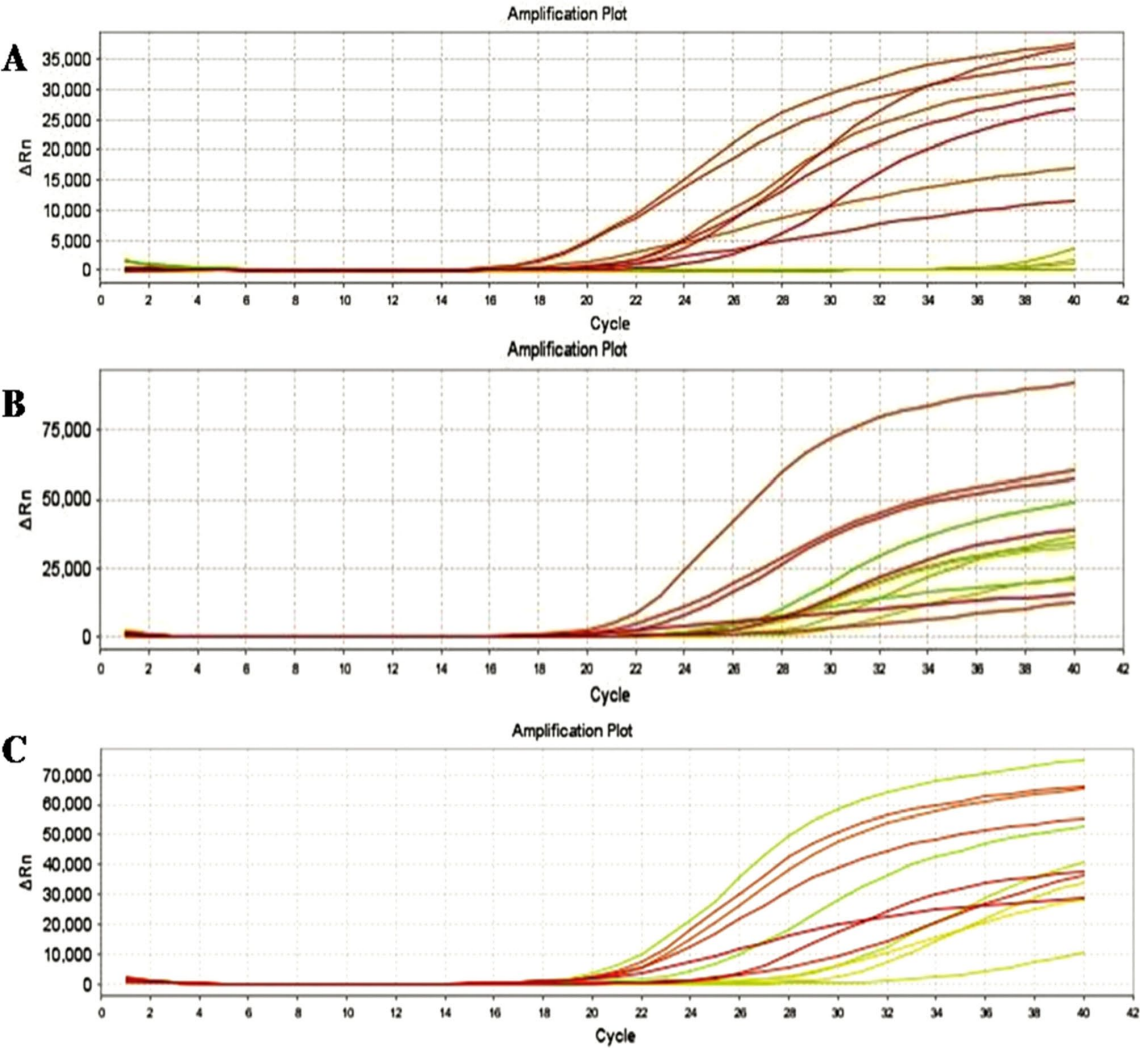
Amplification plot of (**A**) Bcl-2 gene (**B**) BAX gene (**C**) p53 gene in the MCF-7 cell lines

**Fig. 11:**
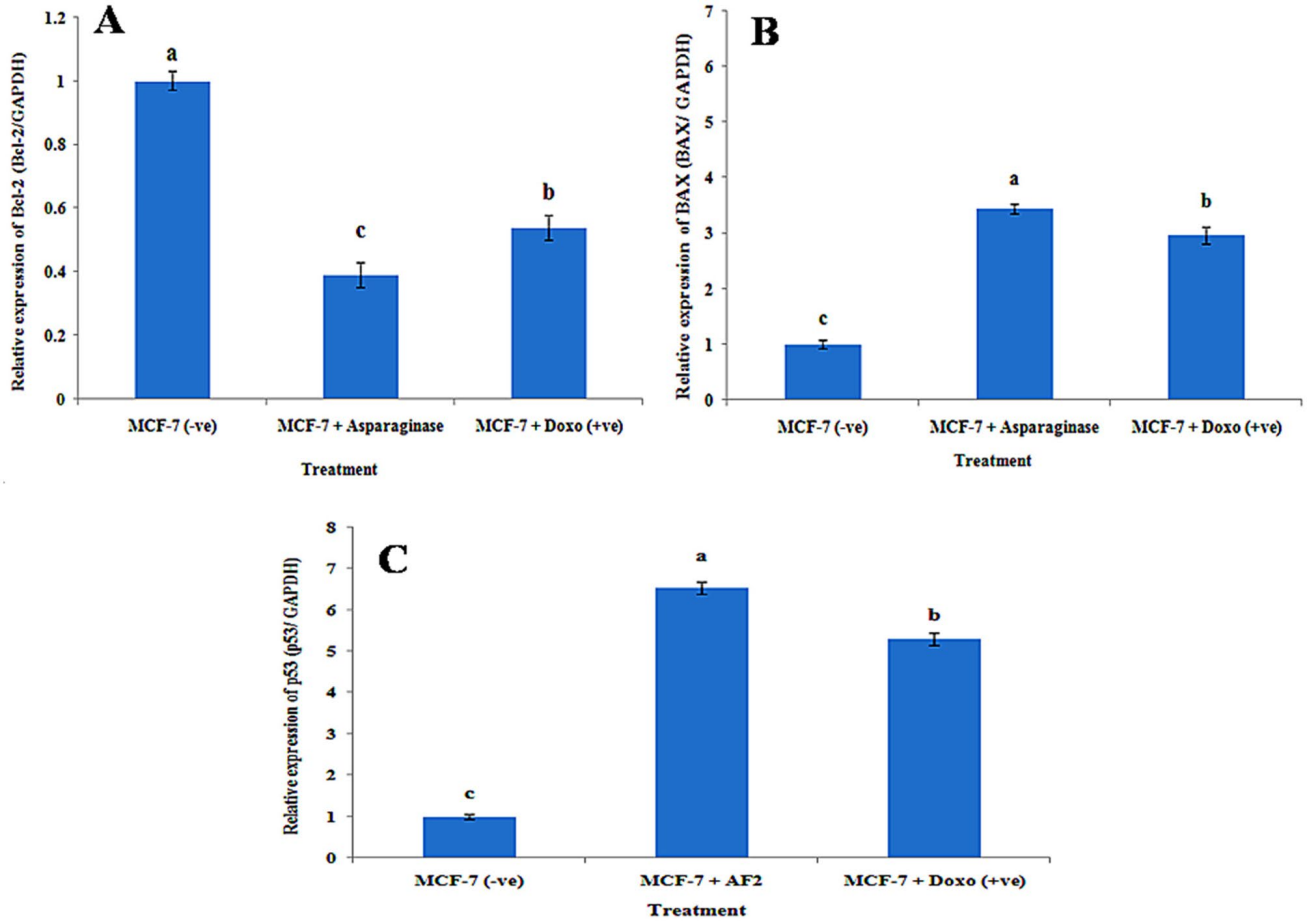
Gene expression of **(A)** BCL-2 **(B)** BAX **(C)** p53 in MCF-7 breast cancer cell lines pretreated with both *Fusarium falciforme* AUMC 16563’ asparaginase and doxorubicin

The antiapoptotic gene BCL-2 was found to be significantly (*p* < 0.05) more highly expressed in negative samples of MCF-7 cells than in those of MCF-7 cells treated with both *Fusarium falciforme* AUMC 16563’ asparaginase and doxorubicin (Fig. 10A). In MCF-7 cells treated with *Fusarium falciforme* AUMC 16563’ asparaginase and doxorubicin, the expression of proapoptotic genes, BAX (Fig. 10B) and p53 (Fig. 10C) was significantly (*p* < 0.05) increased. Expression analysis of BCL-2, p53 and BAX in breast cancer cell lines treated with *F. falciforme* 16563’asparaginase and Doxo showed that the expression levels of the antiapoptotic gene BCL-2 were singnificantly greater in negative samples of breast cancer cell lines than in treated cell lines(Fig. 10A). The expression level of the BCL-2 gene decreased significantly in MCF-7 + Doxo and reached the lowest level in MCF-7 + asparaginase. On the other hand, the expression levels of proapoptotic genes (P53 and BAX) were significantly lower in negative samples of breast cancer cell lines than in treated breast cancer cell lines (Fig. 11B,C). The expression levels of proapoptotic genes (BAX and p53) were significantly graeter in the breast cancer cell lines treated with asparaginase than in the negative control breast cancer cell lines.

### DNA fragmentation

**Fig. 12:**
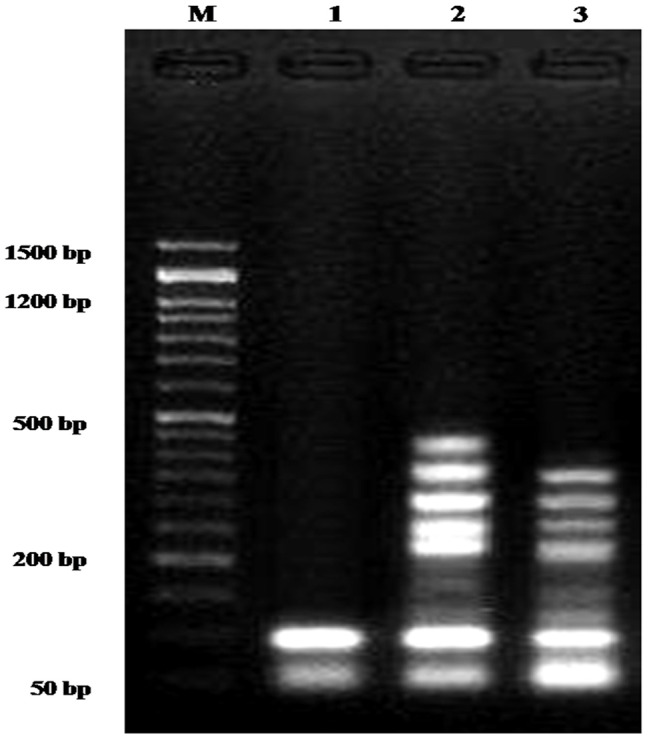
Agarose gel analysis of MCF-7 cancer cell lines exposed to various compounds revealed DNA fragmentation. M: DNA marker; Lane 1: MCF-7 negative control; Lane 2: MCF-7 cells treated with *Fusarium falciforme* AUMC 16563’ asparaginase; Lane 3: MCF-7 cell lines treated with doxorubicin

**Fig. 13:**
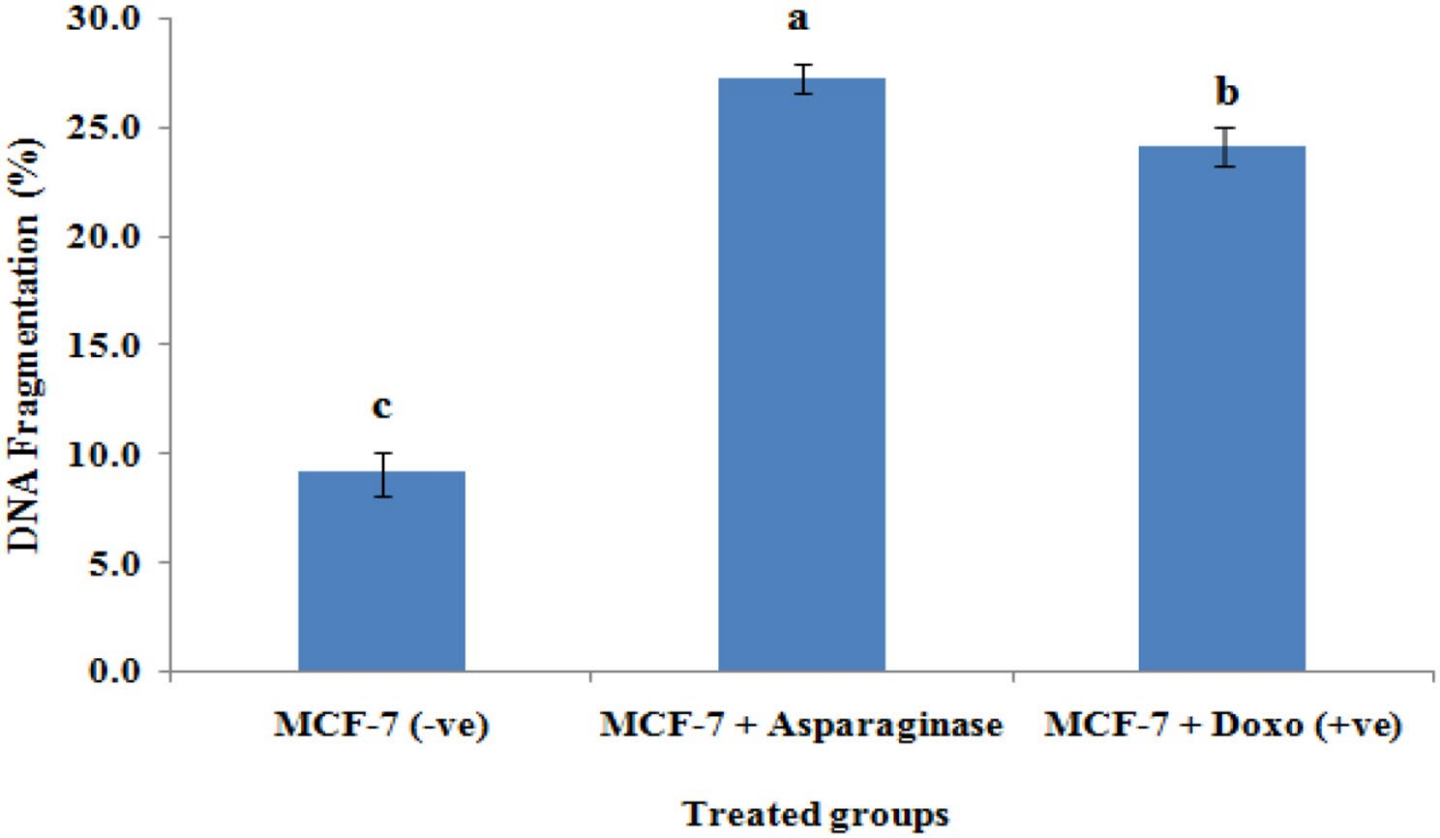
DNA fragmentation identified in MCF-7 cell lines treated with *F. falciforme* AUMC 16563’ asparaginase and doxorubicin (Means with different values between treatments in the same column are substantially different at *p* < 0.05)

The current findings demonstrated that, in comparison with the treated samples, the negative control samples had significantly (*p* < 0.01) lower DNA fragmentation rates (9.1 ± 1.01). In contrast, the DNA fragmentation values of the MCF-7 samples treated with *Fusarium falciforme* AUMC 16563’ asparaginase (27.2 ± 0.69) and the drug doxorubicin (24.1 ± 0.86) were significantly (*p* < 0.01) lower than those of the samples treated with asparaginase alone (Figs. 12, 13).

### In vivo **cytotoxic effects of pure asparaginase in an animal model**

**Fig. 14:**
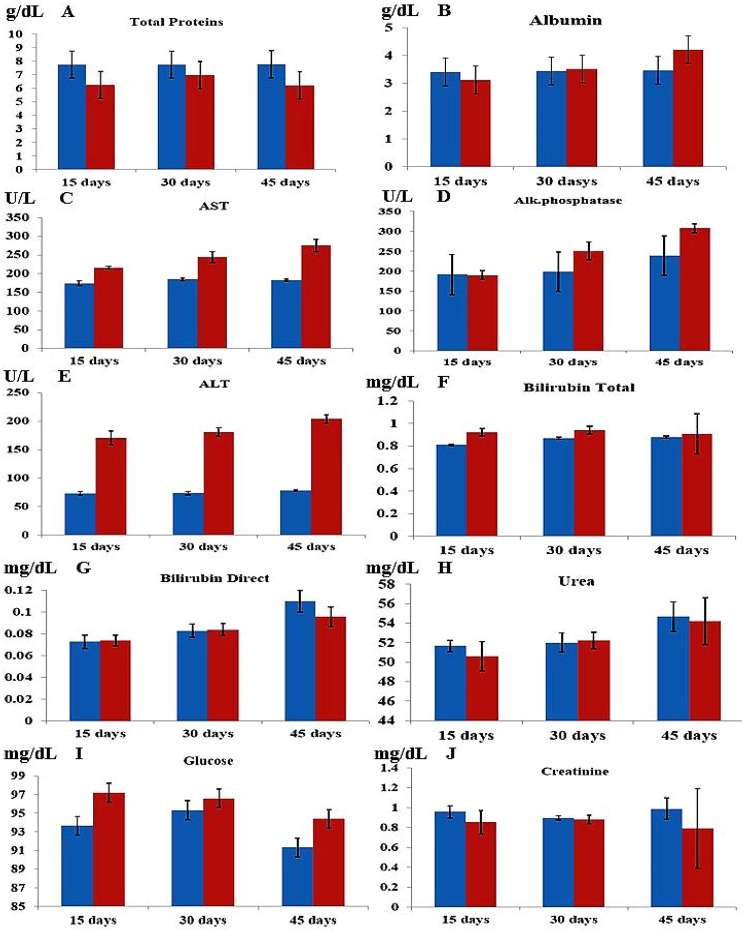
Biochemical parameters of the expremental and control animal models at 15, 30, and 45 days, (**A**) Total proteins (**B**) Albumin (**C**) Alkaline phospahtase (**D**) ALT (**E**) AST (**F**) Bilirubin total (**G**) Bilirubin direct (**H**) Urea (**I**) Creatinine and (**J**) Glucose

**Fig. 15:**
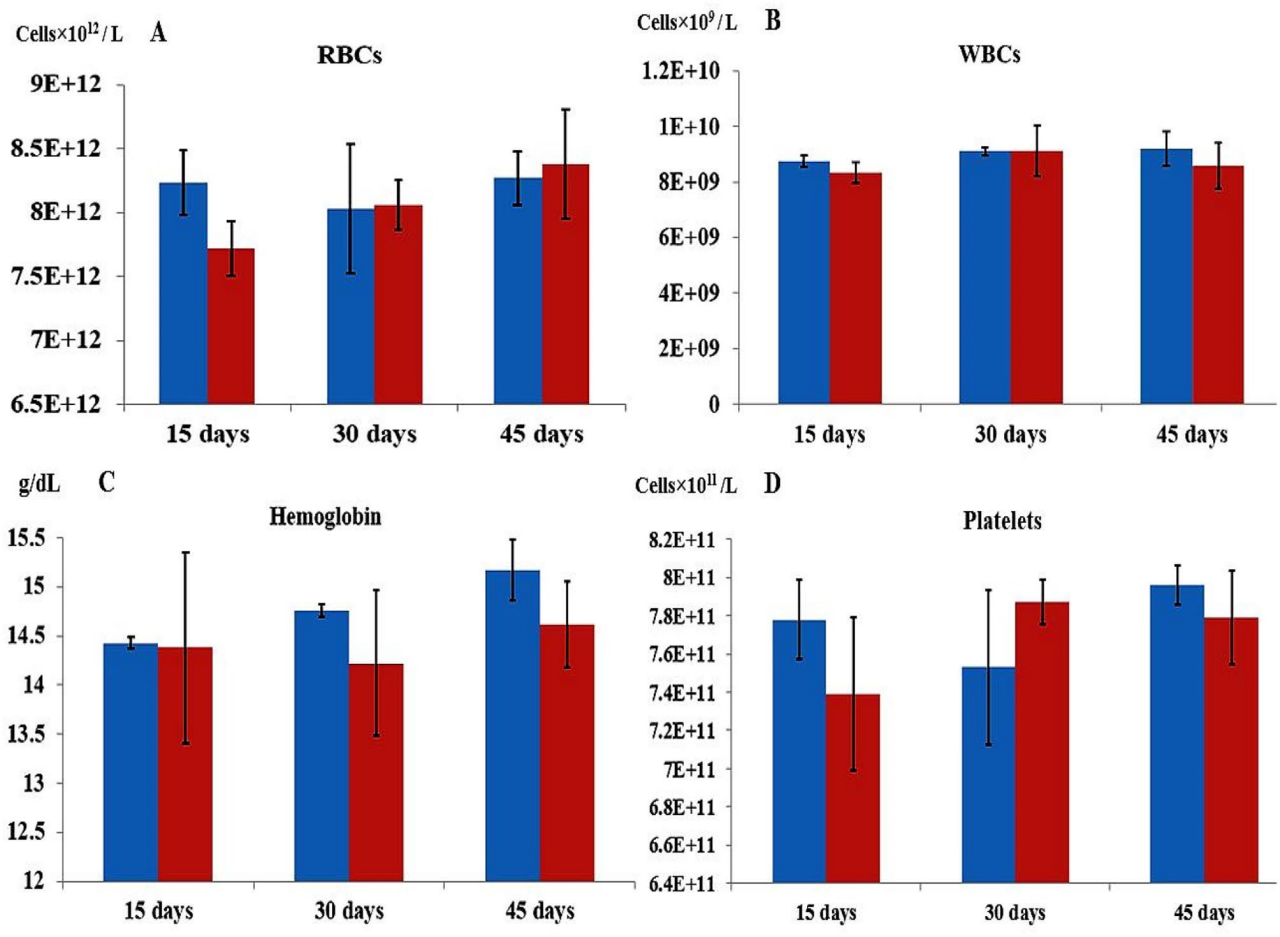
Hematological parameters of the expremental and control animal models at 15, 30, and 45 days, (**A**) RBCs (**B**) WBCs (**C**) Hemoglobin and (**D**) Platelets

The biochemical profiles indicated no impact on glucose levels, other electrolytes, liver function, or kidney function. The total bilirubin level, along with the activities of aspartate aminotransferase (AST) and alanine transaminase (ALT), exhibited slight elevation. The results indicated that the asparaginase from Fusarium falciforme AUMC 16563 had a minimal impact on liver function, with liver impairment likely indicated by AST and ALT markers. All haematological parameters remained within normal ranges throughout the experiment; however, following a 15-day injection of L-asparaginase, white blood cell (WBC), platelet, haemoglobin, and red blood cell counts were slightly lower compared to the control group (preinjection). Furthermore, throughout the experimental periods, the rats remained alive (Tables S1-S3; Figs. 14, 15).

## Discussion

L-Asparaginase is commonly found in plants, animals, and microbes, but it is absent in humans. Microbes serve as a superior source for enzyme production due to their ease of cultivation and manipulation [55]. L-asparaginase, an anti-proliferative enzyme, is found in various fungal species, including *Alternaria*, *Aspergillus*, *Chaetomium*, *Cladosporium*, *Curvularia*, *Drechslera*, *Fusarium*, *Penicillium*, *Pestalotiopsis*, *Phoma*, *Phomopsis*, *Pleurotus*, and *Trichoderma* [56–63].

Continuous exploration of new microbial sources of asparaginase is essential. Therefore, this study aimed at maximizing asparaginase production by *F. falciforme* AUMC 16563, facilitating a better understanding of the independent factors influencing asparaginase production and identifying optimal activity levels and fermentation parameters. *F. falciforme* AUMC 16563 exhibited the highest asparaginase activity of 97.3 U/mL after 5 days at pH 8.0 and 25 °C, utilizing 0.2% glucose and 1.0% asparagine. L-Asparaginase production increased progressively in this study with the growth period, peaking at 120.19 U/mL after 5 days. The current results were consistent with the research conducted by Yadav et al. [64] and Isaac et al. [65], which demonstrated that L-asparaginase derived from *F. oxysporum* and *F. solani* AUMC 8615, respectively reached optimization on the fifth day of incubation. In contrast, *Trichosporon asahii* IBBLA1 exhibited the highest enzyme yield of 20.57 U/mL after 60 h, as documented by Ashok et al. [66]. Asparaginase production from *F. falciforme* AUMC 16563 in this investigation exhibited a progressive rise in activity at temperatures between 20 and 50 °C, peaking at 25 °C.

The pH and temperature of the growth media influence enzyme synthesis by altering the flow of components from the cell membrane [67]. Our findings indicated that the optimal pH for asparaginase production from *F. falciforme* was 8.0, whereas Alrumman et al. [68] found that the perfect pH for asparaginase production from *B. licheniformis* was 6.5. At 30 °C and pH 8.0, utilizing 0.2% glucose and 0.5% ammonium sulphate, our results confirmed the production of asparaginase from *Fusarium solani* AUMC 8615. These conditions represent the optimal parameters for maximal L-asparaginase synthesis through solid-state fermentation, which was achieved on the fifth day of incubation [65].

The incubation temperature is significant since it is regarded as a vital environmental factor for L-asparaginase synthesis by microorganisms, as it regulates microbial growth and, consequently, enzyme production for optimal yield. The incubation temperature assay was conducted at several temperatures, specifically 25, 30, 35, 40, 45, and 50 °C, to evaluate their impact on enzyme synthesis in this investigation. Increased temperatures led to a reduction in enzyme synthesis, which ceased entirely at 50 °C in this study. This may be ascribed to the reduction in the metabolic activity of the microbe, as previously shown [69].

In this study, the highest asparaginase production was achieved after 5 days of incubation. Short incubation periods are generally preferred in the production of enzymes because they are more cost-effective and minimize the risk of enzyme degradation [70]. A study on *F. solani* produced similar findings to ours, demonstrating that five days represented the optimal incubation period [65]. Microorganisms synthesize L-asparaginase at varying optimal incubation durations, as demonstrated by research on *Aspergillus terreus* and *Aspergillus niger* [71], which indicated that incubation exceeding 96 h led to a reduction in enzyme production; 48 h for *Emericella nidulans* [72], and 120 h for *Fusarium* spp. [73]. The maximum L-asparaginase activity recorded by *Fusarium foetens* was 12.83 U/mL, attained after 7 days of incubation at a pH of 8.0 and a temperature of 27.5 °C [74]. *Bacillus halotolerans* ASN9 achieved the highest production of L-asparaginase (9.25 U/mL), at pH 6.0 and 37 °C [75]. Our findings contrast with the previously identified optimal components for the submerged media. The findings of this study concerning optimal fermentation conditions for maximizing L-asparaginase synthesis offer significant insights into the scalability of the process. The enhanced conditions outlined in our findings may promote a more economical and high-yield production of L-asparaginase.

The asparaginase produced from *F. falciforme* AUMC 16563 was purified in this study utilizing two chromatography columns. The use of DEAE-cellulose and Sephacryl S 200 HR columns resulted in a 14.26-fold purification of the enzyme, achieving a yield of 2.38% and a maximum specific activity of 5109.4 U/mg, exceeding values documented in other studies. The current study showcased a highly efficient purification technique that produced enzymes of exceptional activity and purity. L-asparaginase obtained from *P. carotovorum* has undergone purification to a factor of 9.38, resulting in a yield of 23.5% and reaching a maximum specific activity of 202.6 U/mg [76]. *Bacillus halotolerans* ASN9 produces L-asparaginase, resulting in a yield of 24% and a specific activity of 3083 U/mg [75]. The L-asparaginase derived from *F. foetens* underwent a purification process that resulted in a 15.6-fold increase using DEAE-cellulose column chromatography, attaining a yield of 39.89% and a specific activity of 231.38 U/mg [74]. L-asparaginase from *Bacillus licheniformis* PPD37 underwent a purification process achieving a 12.47-fold increase through DEAE-cellulose chromatography. This resulted in an 11.84% yield and an activity level of 7707 U/mg [77]. L-asparaginase obtained from *F. equiseti* AHMF4 underwent purification, achieving a 2.67-fold increase with a yield of 48% and a specific activity of 488.1 U/mg through the use of anion exchange QFF and Sephacryl S200 columns [78]. This analysis indicates that the various purification phases may have improved purity, which is essential for medicinal applications where contaminants could pose harmful effects. This study signifies a notable improvement in the purification techniques for medicinal enzymes through the analysis of purification yields and enzyme recovery rates.

Because of the difference between their native and denatured forms, enzymes are naturally fragile and have limited function. Enzymes’ thermal stability is a key consideration for pharmaceutical applications because these enzymes typically have to have high thermal stability. Based on our findings, the asparaginase was most active and stable at 39 °C, and it maintained 63% of its activity at 42 °C. Despite this, the activity gradually decreased, reaching 35% at 45 °C eventually. Previous research on *Bacillus licheniformis* showed that activity increased from 20 to 35 °C, peaked at 40 °C, and then decreased by about 27.7% at 45 °C [70]. Our results were in line with that work. *Streptomyces gulbargensis* showed similar outcomes; at 40 °C, the enzyme was at its most active [3]. Separate research on the *Fusarium* sp. enzyme found that L-asparaginase was most active between 30 and 40 °C, with reduced activity at higher temperatures and complete inactivation between 50 and 60 °C [79]. Researches on *Fusarium equiseti* [78], *Bacillus aryabhattai* [80], and *Emericella nidulans* [72], all found similar results, suggesting that the improper conformation of the enzymes caused by the denaturation of mesophilic enzymes at high temperatures was to blame for the decrease in enzyme production.

It has been shown that the majority of L-asparaginase enzymes, whether isolated or purified, work best in slightly alkaline environments with pH values between 8 and 10. Our results were supported by the data that showed that enzyme activity was highest at a slightly alkaline pH (pH 8). However, further increases in the alkalinity of the medium may considerably decrease the enzyme’s activity [69, 79]. Comparable outcomes were observed in *Aspergillus fumigatus* [61] and *Phaseolus vulgaris* [81], but in other *Fusarium* species, peak activity occurred at pH 9 [82]. The reason the ideal pH for the enzyme is higher than acidic is because the aspartate that is produced during asparagine hydrolysis has a lower affinity for the active catalytic site of the enzyme. Asparagine is able to bind to the enzyme more effectively because of this. According to Abdelrazek et al. [70], when the pH is acidic, the enzyme breaks down asparagine into aspartic acid. This acid strongly binds to the enzyme’s catalytic site, preventing asparagine from attaching to the enzyme.

This research assessed the impact of certain metal ions and EDTA on the activity of pure asparaginase in this study were demonstrated at a concentration of 5 mM. A notable decrease in enzyme activity was seen in the presence of Mg^2+^, Co^2+^, Mn^2+^, Cu^2+^, Zn^2+^, Ba^2+^, and Cd^2+^. K^+^ demonstrated a markedly stimulatory impact, ranking top among all studied chemicals, followed by Na^+^, Ca^2+^, and Fe^2+^, compared to the control value. The metal chelator EDTA did not influence L-asparaginase activity. The impacts of ions, activators, and inhibitors have been assessed in various research. In relation to this, the addition of 5 mM MgSO_4_ and MnSO_4_ activated the pure L-asparaginase obtained from *A. oryzae* CCT 3940. The activity of L-asparaginase was noted to be slightly reduced in the presence of FeSO_4_, CuSO_4_, KCl, CaCO_3_, and ZnSO_4_. Furthermore, the activity of L-asparaginase was inhibited by ZnSO_4_, CuSO_4_, and CaCl_2_, leading to an approximate reduction of 60% [68]. KCl and NaCl have exhibited stimulatory effects on the asparaginase of *Penicillium cyclopium* [82]. Hg^2+^ reduced the asparaginase activity of *Aspergillus niger* AKV-MKBU by 50%, however Mn^2+^ did not inhibit the enzyme activity. The activity of L-asparaginase obtained from *Fusarium foetens* was enhanced by Mn^2+^, Fe^2+^, and Mg^2+^ [65]. The inhibition of enzyme activity by divalent ions may be due to the chelation of sulfhydryl groups of asparaginase with metal ions [83]. When ions function as an enzyme’s co-substrate, substrate, or co-factor, they can bind (or chelate) with proteins to form complexes that affect protein stability [73]. The asparaginase activity of *Brevibacillus borstelensis* ML12 was significantly increased by CoCl_2_ and KCl, whereas it was considerably reduced by CuSO_4_, FeSO_4_, KNO_3_, and NaNO_3_ [67]. The asparaginase activity in *Fusarium equiseti* AHMF4 augmented by 5.8% and 43.3% following incubation with K^+^ and Mg^2+^ at a concentration of 50 mM; conversely, its activity decreased by 77.5% and 99.1% in the presence of Ca^2+^ and Na^+^ at the identical concentration.

The *K*_m_ and *V*_max_ of the asparaginase produced by *F. falciforme* AUMC 16563 for aspartates, glutamine, aspartic acid, and glutamic acid were determined in this study. The analysis concluded that L-asparagine was the optimal substrate due to its high affinity, evidenced by a *V*_max_ of 128.22 µmol/min and a *K*_m_ of 5.77 × 10^− 2^ mM. The asparaginase from *F. falciforme* 16563 exhibited *K*_m_ and *V*_max_ values of 9.12 mM and 71.66 µmol/min for L-glutamine, 10.1 mM and 62.46 µmol/min for aspartic acid, and 12.2 mM and 49.51 µmol/min for glutamic acid. The *K*_m_ and *V*_max_ of fungal asparaginases have been determined through focused research efforts. The *K*_m_ value of 0.66 mmol/L and *V*max of 313 U/mL were determined for an isolated strain of *Aspergillus oryzae* CCT 3940 L-asparaginase, as reported by Dias et al. [84]. Parashiva, Javaraiah, et al. [74] report that the isolated L-asparaginase from *Fusarium foetens* displayed a *K*_m_ of 23.82 mM and a *V*max of 210.3 U/mL. On the flip side, The *K*_m_ and *V*_max_ of asparaginases from various bacterial strains have been determined. El-Naggar, et al. [52] reported that L-asparaginase obtained from *Streptomyces brollosae* NEAE-115 exhibited a *V*_max_ of 152.6 U/mL/min and a *K*_m_ of 2.139 × 10^− 3^ M when L-asparagine served as the substrate. According to Amer et al., [85], *Weissella paramesenteroides* MN2C2 asparaginase exhibited a *K*_m_ of 4.41 mM and a *V*_max_ of 130.72 U/mL/min. Shafqat et al., [75] revealed that the asparaginase produced by *Bacillus licheniformis* ASN51’ exhibited a *K*_m_ of 0.04 mM and a *V*_max_ of 7750 U. Various species of *Pseudomonas* [86] reported that the asparaginase isolated from the strain PCH199 exhibited a *K*_m_ value of 0.164 mM and a *V*_max_ value of 54.78 U/mg. Mukherjee and Bera [87] reported that the asparaginase produced by *Brevibacillus borstelensis* ML12 exhibited a *K*_m_ value of 0.310 mM and a *V*_max_ value of 121.654 µmol/mL/min. Due to minor methodological variations, comparing enzyme activity data and kinetic characteristics across various studies can often be challenging. It is important to exercise caution when making comparisons.

The results of this investigation demonstrated that *F. falciforme*’ asparaginase displayed limited activity towards L-glutamine while displaying a strong specificity for L-asparagine, which is advantageous in reducing the likelihood of negative effects, such as neurotoxicity. This comparison highlighted the potential benefits of L-asparaginase sourced from *F*. *falciforme* in clinical settings where selectivity is essential for reducing undesirable effects. This selectivity may aid in reducing the inhibition of glutaminase in various tissues. Further in vitro binding and enzymatic studies are necessary to validate and clarify the isoform selectivity suggested by these protein-protein docking assays. The models also serve as a foundation for the structure-based development of modified selectivity profiles for asparaginase variants.

L-asparaginase is a powerful antileukemic medication that most patients administer twice weekly [88–90]. It is necessary to investigate new L-asparaginase enzymes as an alternative to existing ones due to the relative selectivity for the metabolism of cancer tumor cells [90]. Pure asparaginase from *F. falciforme* AUMC 16563 in this study was used to assess its antiproliferative activity against MCF-7, PC-3, HePG-2, and HCT-116 cell lines. Because of its effectiveness in treating many types of cancer, L-asparaginase is a major substance. Our study broadens the applicability of L-asparaginase to prostatic, hepatic, colonic, and breast malignancies, indicating a broader therapeutic potential, building upon the work of Ali et al. [9]., which shown its efficacy in treating lymphoblastic leukemia. The necessity for additional optimization to decrease side effects was further highlighted when comparing the cytotoxic effects found in our investigation with the toxic effects reported by Duval et al. [91]. 

The antiproliferative effects of L-asparaginase on both normal and cancer cells were studied in vitro in this investigation. The current results showed that the percentage of carcinoma cells killed was the same as that of PC-3 cells, with an IC_50_ of 78.6 µg/mL. The HePG-2 cells were rendered cytotoxic at an IC_50_ of 69.6 µg/mL. The HCT-116 cells and MCF-7 cells were both shown to be cytotoxic, with IC_50_ values of 51.5 µg/mL and 32.8 µg/mL, respectively. Consistent with prior studies, our results demonstrated that L-asparaginase enzyme produced by *F. falciforme* AUMC 16563 was harmful to some cancer cells but have little effect on healthy cells.

A study conducted in 2018 revealed that the percentage of cell mortality in cancer cell lines reached 70% and 80% after treatment with crude L-asparaginase sourced from *Aspergillus sydowii* and *F. oxysporum*, respectively [90]. In a separate study, purified L-asparaginase from *F. equiseti* was isolated and demonstrated significant anti-proliferative activity against various malignant cell lines, including cervical epithelioid carcinoma (HeLa), epidermoid larynx carcinoma (Hep-2), hepatocellular carcinoma (HepG-2), colorectal carcinoma (HCT-116), and breast adenocarcinoma (MCF-7), with IC_50_ values of 2.0, 5.0, 12.40, 8.26, and 22.8 µg/mL, respectively. The enzyme showed decreased cytotoxicity to normal cells in vivo, while displaying increased activity, selectivity, and anti-proliferative effects in a dose-dependent manner against malignant cells [78]. Recent research on the cytotoxic effects of *Fusarium* sp. L-asparaginase demonstrated antileukemic activity with an IC_50_ of 50.1 U/mL when tested on RAW2674 leukemic cell lines [79]. The fungal L-asparaginase enzyme has been shown in previous studies to suppress a number of cancer cell lines, including HepG 2, MCF-7, HCT-116, and A-549 [58, 59, 92]. Purified L-asparaginase from the recombinant strain AYA 20 − 1 reduced the viability of HCT-116 cells by 80% and HepG-2 cells by 100%, respectively, according to El-Gendy et al. [58]., whose findings were consistent with our own. Hassan et al. (2018) found that *A. terreus* L-asparaginase had anticancer effects on HCT-116, HepG-2, and MCF-7 cells, according to IC50 values ranging from 3.79 to 12.6 µg/mL. The growth of different tumor cell lines including UACC-62 (melanoma), 786-0 (kidney), NCI-H40 (lung), PC-3 (prostate), NCI-ADR/RES (ovary), and K562 (leukemia) cells was suppressed by purified *A. oryzae* CCT 3940 L-asparaginase [84]. Moreover, L-asparaginase sourced from *A. fumigatus* showed noteworthy anti-proliferative effects on MDA-MB-231 breast cancer cells. Cell death rates of 71%, 87.7%, and 96.5% were achieved with doses of 5, 10, and 20 U of L-asparaginase, respectively [92].

Because it lacks L-asparagine synthetase, the enzyme relies heavily on L-asparaginase to sustain malignant growth, which is why it shows a clear preference for cancer cells. Also, normal cells are unaffected since they have the ability to synthesize L-asparagine through the presence of L-asparagine synthetase, which is present in adequate quantities [7, 79]. The potential cytotoxicity of L-asparaginase against noncancerous cells is a major concern regarding its therapeutic use. Focusing on the enzyme’s antiproliferative actions against cancer cell lines and evaluating its cytotoxicity towards noncancerous cells, the study offers a fair review of its therapeutic potential. Although *F. falciforme* AUMC 16563 showed encouraging antiproliferative effect and increased enzyme synthesis under ideal circumstances, this work resolves a number of issues related to its introduction. According to this research, *F. falciforme* AUMC 16563 has the potential to outperform previously used strains in terms of productivity and efficacy, making it a better candidate for large-scale production. While this study found no changes in glucose, other electrolytes, the liver, or the kidneys when biochemical profiles of pure asparaginase were examined, a small increase in total bilirubin level and an increase in the activities of aspartate aminotransferase (AST) and alanine transaminase (ALT) were noted. Research using AST and ALT indicators also suggested that pure asparaginase might have a small impact on hepatic function.

## Conclusions

In conclusion, our investigation aimed to optimize the production of L-asparaginase from *F. falciforme* AUMC 16563. After passing the enzyme through two chromatography columns, it was considered pure. The molecular weight as well as *K*_m_ and *V*_max_ values of pure asparaginase were determined. Additionally, DNA fragmentation values of MCF-7 cells treated with doxorubicin and pure asparaginase from *F. falciforme* AUMC 16563 were assessed. After seeing considerable cytotoxicity in PC-3, HePG-2, HCT-116, and MCF-7 cells treated with different concentrations of *F. falciforme* AUMC 16563’ pure asparaginase, the IC_50_ was calculated. While the pure asparaginase biochemical profiles showed no changes in glucose, other electrolytes, the liver, or the kidneys, they did show an increase in the activities of aspartate aminotransferase (AST) and alanine transaminase (ALT) and a small rise in total bilirubin. Pure asparaginase may also have a small impact on liver function, according to AST and ALT indicators.

## Electronic supplementary material

Below is the link to the electronic supplementary material.

Supplementary Material 1

## Data Availability

All data related to this manuscript is incorporated in the manuscript and the supplementary material.

## References

[1] Kumar K, Verma N. The various sources and application of L-asparaginase. Asian J Biochem Pharm Res. 2012;2(3):197–205.

[2] Saeed H, Hemida A, El-Nikhely N, Abdel-Fattah M, Shalaby M, Hussein A, et al. Highly efficient *Pyrococcus furiosus* recombinant L-asparaginase with no glutaminase activity: expression, purification, functional characterization, and cytotoxicity on THP-1, A549 and Caco-2 cell lines. Int J Biol Macromol. 2020;156:812–28. 32311402 10.1016/j.ijbiomac.2020.04.080

[3] Amena S, Vishalakshi N, Prabhakar M, Dayanand A, Lingappa K. Production, purification and characterization of L-asparaginase from *Streptomyces gulbargensis*. Brazilian J Microbiol. 2010;41:173–8. 10.1590/S1517-838220100001000025 PMC3768618 24031478

[4] Fontes MG, Silva C, Roldán WH, Monteiro G. Exploring the potential of asparagine restriction in solid cancer treatment: recent discoveries, therapeutic implications, and challenges. Med Oncol. 2024;41(7):176. 38879707 10.1007/s12032-024-02424-3

[5] Asselin B, Rizzari C. Asparaginase pharmacokinetics and implications of therapeutic drug monitoring. Leuk Lymphoma. 2015;56(8):2273–80. 25586605 10.3109/10428194.2014.1003056 PMC4732456

[6] Tong WH, Pieters R, Hop WC, Lanvers-Kaminsky C, Boos J, van der Sluis IM. No evidence of increased asparagine levels in the bone marrow of patients with acute lymphoblastic leukemia during asparaginase therapy. Pediatr Blood Cancer. 2013;60(2):258–61. 22961784 10.1002/pbc.24292

[7] Narta UK, Kanwar SS, Azmi W. Pharmacological and clinical evaluation of L-asparaginase in the treatment of leukemia. Crit Rev Oncol/Hematol. 2007;61(3):208–21. 17011787 10.1016/j.critrevonc.2006.07.009

[8] Radha R, Arumugam N, Gummadi SN. Glutaminase free L-asparaginase from *Vibrio cholerae*: heterologous expression, purification and biochemical characterization. Int J Biol Macromol. 2018;111:129–38. 29307802 10.1016/j.ijbiomac.2017.12.165

[9] Ali U, Naveed M, Ullah A, Ali K, Shah SA, Fahad S, Mumtaz AS. L-asparaginase as a critical component to combat Acute Lymphoblastic Leukaemia (ALL): a novel approach to target ALL. Eur J Pharmacol. 2016;771:199–210. 26698391 10.1016/j.ejphar.2015.12.023

[10] El-Naggar N, El-Ewasy SM, El-Shweihy NM. Microbial L-asparaginase as a potential therapeutic agent for the treatment of acute lymphoblastic leukemia: the pros and cons. Int J Pharmacol. 2014;10(4):182–99.

[11] Husain I, Sharma A, Kumar S, Malik F. Purification and characterization of glutaminase free asparaginase from *Enterobacter cloacae*: in-vitro evaluation of cytotoxic potential against human myeloid leukemia HL-60 cells. PLoS ONE. 2016;11(2):e0148877. 26891220 10.1371/journal.pone.0148877 PMC4758734

[12] Lenicek Krleza J, Katusic Bojanac A, Jakovljevic G. Determination of l-Asparaginase activity and its therapeutic monitoring in children with Hematological Malignancies in a single Croatian Center. Diagnostics. 2024;14(6):623. 38535043 10.3390/diagnostics14060623 PMC10969727

[13] Shi R, Liu Y, Mu Q, Jiang Z, Yang S. Biochemical characterization of a novel L-asparaginase from *Paenibacillus barengoltzii* being suitable for acrylamide reduction in potato chips and mooncakes. Int J Biol Macromol. 2017;96:93–9. 27919811 10.1016/j.ijbiomac.2016.11.115

[14] Verma N, Kumar K, Kaur G, Anand S. L-asparaginase: a promising chemotherapeutic agent. Crit Rev Biotechnol. 2007;27(1):45–62. 17364689 10.1080/07388550601173926

[15] Ciesarová Z, Kukurová K. Impact of l-asparaginase on acrylamide content in fried potato and bakery products. In:Acrylamide in Food. Elsevier. 2024;47391.

[16] Emadi A, Law JY, Strovel ET, Lapidus RG, Jeng LJ, Lee M, et al. Asparaginase *Erwinia chrysanthemi* effectively depletes plasma glutamine in adult patients with relapsed/refractory acute myeloid leukemia. Cancer Chemother Pharmacol. 2018;81:217–22. 29119293 10.1007/s00280-017-3459-6

[17] Asthana N, Azmi W, Microbial. L-asparaginase: A potent antitumour enzyme. 2003.

[18] De Groot N, Lichtenstein N. The action of *Pseudomonas fluorescens* extracts on asparagine and asparagine derivatives. Biochim Biophys Acta. 1960;40:99–110. 13829573 10.1016/0006-3002(60)91319-6

[19] Rowley B, Wriston JC Jr. Partial purification and antilymphoma activity of *Serratia marcescens* L-asparaginase. Biochem Biophys Res Commun. 1967;28(2):160–5. 10.1016/0006-291x(67)90423-8 5340729

[20] Kozak M, Jurga S. A comparison between the crystal and solution structures of *Escherichia coli* asparaginase II. Acta Biochim Pol. 2002;49(2):509–13. 12362993

[21] Wade BA. Studies on the biology of the west Indian beach clam, *Donax denticulatus* Linne. 2. Life-history. Bull Mar Sci. 1968;18(4):876–901.

[22] Tosa T, Sano R, Yamamoto K, Nakamura M, Ando K, Chibata I. L-Asparaginase from *Proteus vulgaris*. Appl Microbiol. 1971;22(3):387–92. 5000866 10.1128/am.22.3.387-392.1971 PMC376319

[23] Sarquis MIM, Oliveira EMM, Santos AS, Costa GL. Production of L-asparaginase by filamentous fungi. Mem Inst Oswaldo Cruz. 2004;99:489–92. 15543411 10.1590/s0074-02762004000500005

[24] Qeshmi FI, Homaei A, Fernandes P, Javadpour S. Marine microbial L-asparaginase: biochemistry, molecular approaches and applications in tumor therapy and in food industry. Microbiol Res. 2018;208:99–112. 29551216 10.1016/j.micres.2018.01.011

[25] da Cunha MC, dos Santos Aguilar JG, de Melo RR, Nagamatsu ST, Ali F, de Castro RJS, Sato HH. Fungal L-asparaginase: strategies for production and food applications. Food Res Int. 2019;126:108658. 31732030 10.1016/j.foodres.2019.108658

[26] Nguyen HA, Su Y, Zhang JY, Antanasijevic A, Caffrey M, Schalk AM, et al. A novel l-asparaginase with low l-glutaminase coactivity is highly efficacious against both T-and B-cell acute lymphoblastic leukemias in vivo. Cancer Res. 2018;78(6):1549–60. 29343523 10.1158/0008-5472.CAN-17-2106 PMC5856643

[27] Radadiya A, Zhu W, Coricello A, Alcaro S, Richards NG. Improving the treatment of acute lymphoblastic leukemia. Biochemistry. 2020;59(35):3193–200. 32786406 10.1021/acs.biochem.0c00354 PMC7497903

[28] Sobat M, Asad S, Kabiri M, Mehrshad M. Metagenomic discovery and functional validation of L-asparaginases with anti-leukemic effect from the Caspian Sea. Iscience. 2021;24(1). 10.1016/j.isci.2020.101973 PMC7797908 33458619

[29] Imada A, Igarasi S, Nakahama K, Isono M. Asparaginase and glutaminase activities of micro-organisms. Microbiology. 1973;76(1):85–99. 10.1099/00221287-76-1-85 4723072

[30] Gulati R, Saxena R, Gupta R. A rapid plate assay for screening l-asparaginase producing microorganisms. Lett Appl Microbiol. 1997;24(1):23–6. 9024001 10.1046/j.1472-765x.1997.00331.x

[31] Moubasher AH, Ismail MA, Al-Bedak OA, Mohamed RA. *RamophialoChlamydosporaospora*, a new species from an alkaline lake of Wadi-El-Natron, Egypt. Asian J Mycol. 2019;2(1):110–7.

[32] Al-Bedak OA, Moubasher AH. *AspergGaarensisrensis*, a new addition to secircumdatiumdati from soil of Lake El-Gaar in Wadi-El-Natron, Egypt. Stud Fungi. 2020;5(1):59–65.

[33] White T. Amplification and direct sequencing of fungal ribosomal RNA genes for phylogenetics. PCR Protocols: A guide to methods and applications/Academic Press, Inc.; 1990.

[34] Katoh K, Standley DM. MAFFT multiple sequence alignment software version 7: improvements in performance and usability. Mol Biol Evol. 2013;30(4):772–80. 23329690 10.1093/molbev/mst010 PMC3603318

[35] Criscuolo A, Gribaldo S. BMGE (Block Mapping and gathering with Entropy): a new software for selection of phylogenetic informative regions from multiple sequence alignments. BMC Evol Biol. 2010;10:1–21. 20626897 10.1186/1471-2148-10-210 PMC3017758

[36] Kumar S, Stecher G, Li M, Knyaz C, Tamura K. MEGA X: molecular evolutionary genetics analysis across computing platforms. Mol Biol Evol. 2018;35(6):1547–9. 29722887 10.1093/molbev/msy096 PMC5967553

[37] Felsenstein J. Confidence limits on phylogenies: an approach using the bootstrap. Evolution. 1985;39(4):783–91. 28561359 10.1111/j.1558-5646.1985.tb00420.x

[38] Posada D, Crandall KA. MODELTEST: testing the model of DNA substitution. Bioinf (Oxford England). 1998;14(9):817–8. 10.1093/bioinformatics/14.9.817 9918953

[39] Moharram AM, Zohri A-NA, Hesham AE-L, Abdel-Raheam HE, Al-Ameen Maher M, Al-Bedak OA. Production of cold-active pectinases by three novel *Cladosporium* species isolated from Egypt and application of the most active enzyme. Sci Rep. 2022;12(1):15599. 36114347 10.1038/s41598-022-19807-z PMC9481535

[40] Matsumoto H, Haniu H, Komori N. Determination of protein molecular weights on SDS-PAGE. Electrophoretic Sep Proteins: Methods Protocols. 2019:101–5. 10.1007/978-1-4939-8793-1_10 30426411

[41] Lineweaver H, Burk D. The determination of enzyme dissociation constants. J Am Chem Soc. 1934;56(3):658–66.

[42] Mosmann T. Rapid colorimetric assay for cellular growth and survival: application to proliferation and cytotoxicity assays. J Immunol Methods. 1983;65(1–2):55–63. 6606682 10.1016/0022-1759(83)90303-4

[43] El-Menshawi BS, Fayad W, Mahmoud K, El-Hallouty SM, El-Manawaty M. Screening of natural products for therapeutic activity against solid tumors. 2010. 21046978

[44] Thabrew MI, Hughes RD, McFarlane IG. Screening of hepatoprotective plant components using a HepG2 cell cytotoxicity assay. J Pharm Pharmacol. 1997;49(11):1132–5. 9401951 10.1111/j.2042-7158.1997.tb06055.x

[45] Yawata A, Adachi M, Okuda H, Naishiro Y, Takamura T, Hareyama M, Takayama S, Reed JC, Imai K. Prolonged cell survival enhances peritoneal dissemination of gastric cancer cells. Oncogene. 1998;16(20):2681–6. 9632144 10.1038/sj.onc.1201792

[46] Gibb RK, Taylor DD, Wan T, O’Connor DM, Doering DL, Gerçel-Taylor Ç. Apoptosis as a measure of chemosensitivity to cisplatin and taxol therapy in ovarian cancer cell lines. Gynecol Oncol. 1997;65(1):13–22. 9103385 10.1006/gyno.1997.4637

[47] Linjawi SA, Salem LM, Khalil WK. Jatropha curcas L. kernel prevents benzene induced clastogenicity, gene expression alteration and apoptosis in liver tissues of male rats. Indian Journal of Experimental Biology. 201755(04):225–234.

[48] Brito A, Abrantes A, Pinto-Costa C, Gomes A, Mamede A, Casalta-Lopes J, et al. Hepatocellular carcinoma and chemotherapy: the role of p53. Chemotherapy. 2013;58(5):381–6. 10.1159/000343656 23257706

[49] Khalil WKB, Zarouk W, Eldeen GN, Ramadan A, Fayez A, Esmaiel N, et al. Apoptosis, reactive oxygen species and DNA damage in familial Mediterranean fever patients. Gene Rep. 2019;14:76–80.

[50] Ramadan MA, Shawkey AE, Rabeh MA, Abdellatif AO. Expression of P53, BAX, and BCL-2 in human malignant melanoma and squamous cell carcinoma cells after tea tree oil treatment in vitro. Cytotechnology. 2019;71:461–73. 30599074 10.1007/s10616-018-0287-4 PMC6368524

[51] Refaie AA, Ramadan A, Sabry NM, Khalil WK, Mossa A-TH. Over-gene expression in the apoptotic, oxidative damage and liver injure in female rats exposed to butralin. Environ Sci Pollut Res. 2020;27:31383–93. 10.1007/s11356-020-09416-6 32488703

[52] El-Naggar NE-A, Deraz SF, El-Ewasy SM, Suddek GM. Purification, characterization and immunogenicity assessment of glutaminase free L-asparaginase from *Streptomyces brollosae* NEAE-115. BMC Pharmacology and Toxicology. 2018;19:1–15. 10.1186/s40360-018-0242-1 PMC6108126 30139388

[53] Rodrigues MA, Pimenta MV, Costa IM, Zenatti PP, Migita NA, Yunes JA, et al. Influence of lysosomal protease sensitivity in the immunogenicity of the antitumor biopharmaceutical asparaginase. Biochem Pharmacol. 2020;182:114230. 32979352 10.1016/j.bcp.2020.114230

[54] St L, Wold S. Analysis of variance (ANOVA). Chemometr Intell Lab Syst. 1989;6(4):259–72.

[55] Kumar DS, Sobha K. L-asparaginase from microbes: a comprehensive review. Adv Bioresearch. 2012;3(4).

[56] Prihanto A, Caisariyo I, Pradarameswari K. *Aspergillus* sp. as a potential producer for L-Asparaginase from mangrove (*Avicennia germinans*). In: IOP Conference Series: Earth and Environmental Science. 2019; 012101.

[57] Mervat MA, Nageh A, Taher T, Fareed H. Production, purification and characterization of L-asparaginase from marine endophytic *aspergillus* sp. ALAA-2000 under submerged and solid state fermentation. J Microb Biochem Technol. 2015;7(3):165–72.

[58] El-Gendy MMAA, Al-Zahrani SHM, El-Bondkly AMA. Construction of potent recombinant strain through intergeneric protoplast fusion in endophytic fungi for anticancerous enzymes production using rice straw. Appl Biochem Biotechnol. 2017;183:30–50. 28205049 10.1007/s12010-017-2429-0

[59] El-Gendy MMAA, Yahya SM, Hamed AR, Soltan MM, El-Bondkly AMA. Phylogenetic analysis and biological evaluation of marine endophytic fungi derived from Red Sea sponge Hyrtios Erectus. Appl Biochem Biotechnol. 2018;185:755–77. 29327320 10.1007/s12010-017-2679-x

[60] Bedaiwy M. Production, optimization, and anti-cancer activity of L-asparaginase of *Pleurotus ostreatus* under solid state fermentation. Int J Biosci. 2019;14(2):251–63.

[61] Benchamin D, Sreejai R, Athira L, Jensy Roshan F, Sujitha S, Kurup BS. Production and characterization of L-Asparaginase isolated from *Aspergillus Fumigatus*. Pharma Innov J. 2019a;8(3):220–3.

[62] Pallem C. Solid-state fermentation of corn husk for the synthesis of asparaginase by *Fusarium oxysporum*. Asian J Pharm Pharmacol. 2019;5(4):678–81.

[63] Pallem C. Utilization of wheat straw for the production of asparaginase in solid-state fermentation. 2019.

[64] Yadav N, Sarkar S. Production of L-asparaginase by *Fusarium oxysporum* using submerged fermentation. Int J Pharm Sci Invent. 2014;3(6):32–40.

[65] Isaac G, Abu-Tahon M. Production of extracellular anti-leukemic enzyme L-asparaginase from *Fusarium Solani* AUMC 8615 grown under solid-state fermentation conditions: purification and characterization of the free and immobilized enzyme. Egypt J Bot. 2016;56(3):799–816.

[66] Ashok A, Doriya K, Rao JV, Qureshi A, Tiwari AK, Kumar DS. Microbes producing L-asparaginase free of glutaminase and urease isolated from extreme locations of Antarctic soil and moss. Sci Rep. 2019;9(1):1423. 30723240 10.1038/s41598-018-38094-1 PMC6363723

[67] Castro D, Marques ASC, Almeida MR, de Paiva GB, Bento HB, Pedrolli DB, Freire MG, Tavares AP, Santos-Ebinuma VC. L-asparaginase production review: bioprocess design and biochemical characteristics. Appl Microbiol Biotechnol. 2021;105:4515–34. 34059941 10.1007/s00253-021-11359-y

[68] Alrumman S, Mostafa Y, Al-Izran KA, Alfaifi M, Taha T, Elbehairi S. Production and anticancer activity of an L-asparaginase from *Bacillus licheniformis* isolated from the Red Sea, Saudi Arabia. Sci Rep. 2019;9(1):3756. 30842557 10.1038/s41598-019-40512-x PMC6403232

[69] El-Hefnawy M, Attia M, El-Hofy M, Ali SM. Optimization production of L asparaginase by locally isolated filamentous fungi from Egypt. Curr Sci Int. 2015;4(3):330–41.

[70] Abdelrazek NA, Elkhatib WF, Raafat MM, Aboulwafa MM. Experimental and bioinformatics study for production of L-asparaginase from *Bacillus licheniformis*: a promising enzyme for medical application. AMB Express. 2019;9(1):39. 30900037 10.1186/s13568-019-0751-3 PMC6428875

[71] Varalakshmi V, Raju KJ. Optimization of l-asparaginase production by *aspergillus terreus* mtcc 1782 using bajra seed flour under solid state fermentation. Int J Res Eng Technol. 2013;2(09):121–9.

[72] Jayaramu M, Hemalatha N, Rajeshwari C, Siddalingeshwara K, Mohsin S, Dutt PS. A novel approach for detection, confirmation and optimization of L-asparaginase from *Emericella nidulans*. J Curr Pharma Res. 2010;1(1):20.

[73] Murali T. L-asparaginase from marine derived fungal endophytes of seaweeds. Mycosphere. 2011:147–55.

[74] Parashiva J, Nuthan BR, Bharatha M, Praveen R, Tejashwini P, Satish S. Response surface methodology based optimized production, purification, and characterization of L-asparaginase from *Fusarium foetens*. World J Microbiol Biotechnol. 2023;39(9):252. 37442849 10.1007/s11274-023-03684-3

[75] Shafqat I, Shahzad S, Yasmin A, Almusallam SY, Al-Megrin WAI. Efficient Production, Purification and Characterization of Therapeutically Significant L-Asparaginase from *Bacillus licheniformis* ASN51. Polish Journal of Environmental Studies. 2023;32(5).

[76] Do TT, Do TP, Nguyen TN, Nguyen TC, Vu TTP, Nguyen TGA. Nanoliposomal L-Asparaginase and its Antitumor activities in Lewis Lung Carcinoma Tumor‐Induced BALB/c mice. Adv Mater Sci Eng. 2019;2019(1):3534807.

[77] Patel P, Patel A, Agarwal-Rajput R, Rawal R, Dave B, Gosai H. Characterization, anti-proliferative activity, and bench-scale production of novel pH-stable and thermotolerant l-asparaginase from *Bacillus licheniformis* PPD37. Applied Biochemistry and Biotechnology. 2023;195(5):3122–3141. 10.1007/s12010-022-04281-0 36564676

[78] El-Gendy MMAA, Awad MF, El-Shenawy FS, El-Bondkly AMA. Production, purification, characterization, antioxidant and antiproliferative activities of extracellular L-asparaginase produced by *Fusarium equiseti* AHMF4. Saudi J Biol Sci. 2021;28(4):2540–8. 33911966 10.1016/j.sjbs.2021.01.058 PMC8071902

[79] Yousef A SA. Fusarium sp. L-asparaginases: purification, characterization, and potential assessment as an antileukemic chemotherapeutic agent. Environ Sci Pollut Res. 2022;29(8):11243–54. 10.1007/s11356-021-16175-5 34532809

[80] Singh Y, Srivastava S. L-asparaginase production by a new isolate *Bacillus aryabhattai* strain ITBHU02 in solid state culture. In: 1st International Conference on Biosciences and Bioengineering: A collaborative Approach. 2012;150–168.

[81] Mohamed SA, Elshal MF, Kumosani TA, Aldahlawi AM, Basbrain TA, Alshehri FA, Choudhry H. L-asparaginase isolated from *phaseolus vulgaris* seeds exhibited potent anti-acute lymphoblastic leukemia effects in-vitro and low immunogenic properties in-vivo. Int J Environ Res Public Health. 2016;13(10):1008. 27754445 10.3390/ijerph13101008 PMC5086747

[82] Asha A, Pallavi B. Production, purification and characterization of extracellular L-asparaginase having antineoplastic activity from *Fusarium* Sp. J Adv Res Biol Sci. 2012;4(4):293–301.

[83] Kumar S, Darnal S, Patial V, Kumar V, Singh D. Molecular characterization of a stable and robust L-asparaginase from *Pseudomonas* sp. PCH199: evaluation of cytotoxicity and acrylamide mitigation potential. Fermentation. 2022;8(10):568.

[84] Dias FFG, Ruiz ALTG, Della Torre A, Sato HH. Purification, characterization and antiproliferative activity of L-asparaginase from *Aspergillus oryzae* CCT 3940 with no glutaminase activity. Asian Pac J Trop Biomed. 2016;6(9):785–94.

[85] Amer M, Atwa N, Eldiwany A, Elgammal E, Dawoud I, Rashad F. Anticancer and antioxidant activities of l-asparaginase produced by local *Weissella paramesenteroides* MN2C2 strain. Egypt J Chem. 2022;65(7):409–19.

[86] Darnal S, Patial V, Kumar V, Kumar S, Kumar V, Padwad YS, Singh D. Biochemical characterization of extremozyme L-asparaginase from *Pseudomonas* sp. PCH199 for therapeutics. AMB Express. 2023;13(1):22. 36828987 10.1186/s13568-023-01521-2 PMC9958223

[87] Mukherjee R, Bera D. Immobilization and biochemical characterization of purified L-Asparaginase produced by *Brevibacillus borstelensis* ML12. Eur J Sci Res Reviews. 2024;1(1):69–69.

[88] Graham ML. Pegaspargase: a review of clinical studies. Adv Drug Deliv Rev. 2003;55(10):1293–302. 14499708 10.1016/s0169-409x(03)00110-8

[89] Oza VP, Parmar PP, Kumar S, Subramanian R. Anticancer properties of highly purified L-asparaginase from *Withania somnifera* L. against acute lymphoblastic leukemia. Appl Biochem Biotechnol. 2010;160:1833–40. 19448978 10.1007/s12010-009-8667-z

[90] Ali D, Ouf S, Eweis M, Solieman D. Optimization of L-asparaginase production from some filamentous fungi with potential pharmaceutical properties. Egypt J Bot. 2018;58(3):355–69.

[91] Duval M, Suciu S, Ferster A, Rialland X, Nelken B, Lutz P, et al. Comparison of *Escherichia coli*–asparaginase with *Erwinia*-asparaginase in the treatment of childhood lymphoid malignancies: results of a randomized European Organisation for Research and Treatment of Cancer—Children’s Leukemia Group phase 3 trial. Blood J Am Soc Hematol. 2002;99(8):2734–9. 10.1182/blood.v99.8.2734 11929760

[92] Benchamin D, Sreejai R, Sujitha S, Albert C, Rishad K. Anti-proliferative activity of L-Asparaginase enzyme from fungi on breast cancer. J Pharmacognosy Phytochemistry. 2019b;8(1):407–10.

