# Supplementary material for: L-asparaginase from the novel Fusarium falciforme AUMC 16563: extraction, purification, characterization, and cytotoxic effects on PC-3, HePG-2, HCT-116, and MCF-7 cell lines
Source: BMC Microbiol. 2025 Mar 17;25:145. doi: 10.1186/s12866-025-03833-8 (PMC11912728; doi:10.1186/s12866-025-03833-8)
Supplement: Supplementary file 1 — Supplementary Material 1 [file 12866_2025_3833_MOESM1_ESM.docx]

L-asparaginase from the novel *Fusarium falciforme* AUMC 16563: Extraction, purification, characterization, and cytotoxic effects on PC-3, HePG-2, HCT-116, and MCF-7 cell lines

Abdullah Abobakr Saleh^1, 2*^, Hamdy M. El-Aref^1, 3*^, Azza M. Ezzeldin^4^, Rania M. Ewida^5^, Osama A. M. Al-Bedak^6,7^

^1^Molecular Biology Researches & Studies Institute, Assiut University, Egypt; [Abdullah.abobakr@science.aun.edu.eg](mailto:Abdullah.abobakr@science.aun.edu.eg) (0000-0002-8597-3842)

^2^South Egypt Cancer Institute, Department of Clinical Pathology and Hematological Malignancies, Assiut University, 71511, Egypt;

^3^Department of Genetics, Faculty of Agriculture, Assiut University, Assiut 71511, Egypt; [hmelaref_2016@aun.edu.eg](mailto:hmelaref_2016@aun.edu.eg) (0000-0002-4167-6221)

^4^Clinical Pathology Department, Faculty of Medicine, Assiut University, 71511, Egypt;

[azzam80@aun.edu.eg](mailto:azzam80@aun.edu.eg)

^5^Food Hygiene, Safety and Technology Department, Faculty of Veterinary Medicine, New Valley University, El-Kharga, 72511, Egypt; [r_ewida@vet.nvu.edu.eg](mailto:r_ewida@vet.nvu.edu.eg) (0000-0003-2110-6851)

^6^Assiut University Mycological Centre, Assiut University, Assiut 71511, Egypt; [osamaalbedak@science.au.edu.eg](mailto:osamaalbedak@science.au.edu.eg) (0000-0003-0465-619X).

^7^ ERU Science & Innovation Center of Excellence, Egyptian Russian University, Badr city, 11829, Egypt

^*^Correspondences: [hmelaref_2016@aun.edu.eg -](mailto:hmelaref_2016@aun.edu.eg%20-) [Abdullah.abobakr@science.aun.edu.eg](mailto:Abdullah.abobakr@science.aun.edu.eg)

**
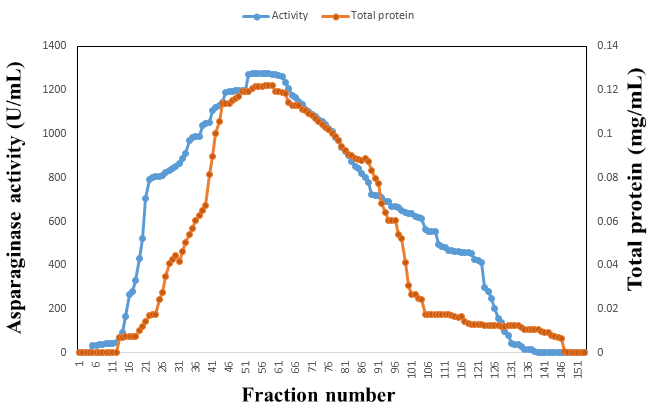
**

**Fig. S1.** Fractional purification pattern of the L- asparaginase produced by *F. falciforme* applying DEAE-cellulose column chromatography.

**
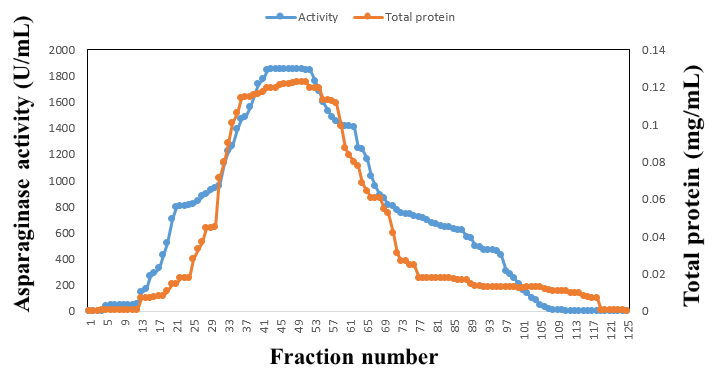
**

**Fig. S2.** Fractional purification pattern of the L- asparaginase produced by *F. falciforme* applying Sephacryl S-200HR column chromatography.

**Table S1:** Difference in biochemical and hematological parameters between the control group and tested group on normal mice after 15 days

| **Variables** | **Control (Mean ±SD)** | **Tested group (Mean ±SD)** | **P value** |
| --- | --- | --- | --- |
| Total proteins | 7.75±0.15 | 6.24±0.09 | **<0.001^**^** |
| Albumin | 3.4±0.015 | 3.11±0.02 | **<0.001^**^** |
| Alk. Phosphatase | 191.67±5.5 | 189.8±4.76 | 0.629^NS^ |
| S.GOT (AST) | 174.67±6.03 | 216.2±3.96 | **<0.001^**^** |
| S.GPT (ALT) | 73.33±3.21 | 170.8±12.38 | **<0.001^**^** |
| Bilirubin: Total | 0.81±0.006 | 0.924±0.033 | **0.001^**^** |
| Bilirubin Direct | 0.073±0.006 | 0.074±0.005 | 0.875^NS^ |
| Urea | 51.67±0.58 | 50.6±1.52 | 0.298^NS^ |
| Creatinine | 0.96±0.06 | 0.854±0.12 | 0.214^NS^ |
| Serum Glucose | 93.67±6.66 | 97.2±4.1 | 0.379^NS^ |
| RBC | (8.23×10^12^)±(2.52 ×10^11^) | (7.72×10^12^)±(2.17 ×10^11^) | **0.022^*^** |
| WBC | (8.73×10^9^)±(2.08 ×10^8^) | (8.32×10^9^)±(3.77 ×10^8^) | 0.137^NS^ |
| Hemoglobin | 14.43±0.06 | 14.38±0.97 | 0.929^NS^ |
| Platelets | (7.78×10^11^)±(2.05 ×10^10^) | (7.39×10^11^)±(4.03 ×10^10^) | 0.183^NS^ |

Data expressed as Mean ±SD.^*^, ^**^ and NS indicate p < 0.05, p < 0.01 and not significant, respectively.

**Table S2:** Difference in biochemical and hematological parameters between the control group and tested group on normal mice after 30 days

| **Variables** | **Control (Mean ±SD)** | **Tested group (Mean ±SD)** | **P value** |
| --- | --- | --- | --- |
| Total proteins | 7.75±0.12 | 6.96±0.22 | **0.001^**^** |
| Albumin | 3.44±0.075 | 3.51±0.27 | 0.701^NS^ |
| Alk. Phosphatase | 198.33±4.04 | 250.8±22.05 | **0.007^**^** |
| S.GOT (AST) | 185±3.6 | 244.4±13.88 | **<0.001^**^** |
| S.GPT (ALT) | 73.67±3.21 | 181.2±7.46 | **<0.001^**^** |
| Bilirubin: Total | 0.87±0.01 | 0.94±0.035 | **0.016^*^** |
| Bilirubin Direct | 0.083±0.006 | 0.084±0.0055 | 0.875^NS^ |
| Urea | 52±1 | 52.2±0.84 | 0.77^NS^ |
| Creatinine | 0.9±0.021 | 0.884±0.043 | 0.505^NS^ |
| Serum Glucose | 95.33±7.37 | 90.6±10.06 | 0.510^NS^ |
| RBC | (8.03×10^12^) ± (5.03 ×10^11^) | (8.06×10^12^) ± (1.95 ×10^11^) | 0.916^NS^ |
| WBC | (9.1×10^9^) ± (1.53 ×10^8^) | (9.1×10^9^) ± (9.01 ×10^8^) | 0.981^NS^ |
| Hemoglobin | 14.76±0.06 | 14.22±0.74 | 0.262^NS^ |
| Platelets | (7.53×10^11^) ± (4.06 ×10^10^) | (7.87×10^11^) ± (1.15 ×10^10^) | 0.115^NS^ |

Data expressed as Mean ±SD.^*^, ^**^ and NS indicate p < 0.05, p < 0.01 and not significant, respectively.

**Table S3:** Difference in biochemical and hematological parameters between the control group and tested group on normal mice after 45 days

| **Variables** | **Control (Mean ±SD)** | **Tested group (Mean ±SD)** | **P value** |
| --- | --- | --- | --- |
| Total proteins | 7.76±0.15 | 6.22±0.08 | **<0.001^**^** |
| Albumin | 3.46±0.05 | 4.2±0.084 | **<0.001^**^** |
| Alk. Phosphatase | 239±4.58 | 307.8±11.12 | **<0.001^**^** |
| S.GOT (AST) | 182.33±3.21 | 275.6±16.62 | **<0.001^**^** |
| S.GPT (ALT) | 78.67±1.15 | 204.2±7.16 | **<0.001^**^** |
| Bilirubin: Total | 0.88±0.01 | 0.908±0.18 | 0.05^NS^ |
| Bilirubin Direct | 0.11±0.01 | 0.096±0.009 | 0.085^NS^ |
| Urea | 54.67±1.53 | 54.2±2.39 | 0.775^NS^ |
| Creatinine | 0.99±0.11 | 0.793±0.4 | 0.448^NS^ |
| Serum Glucose | 91.33±6.66 | 94.4±13.4 | 0.73^NS^ |
| RBC | (8.27×10^12^)±(2.1 ×10^11^) | (8.38×10^12^)±(4.27 ×10^11^) | 0.688^NS^ |
| WBC | (9.2×10^9^)±(6.1 ×10^8^) | (8.56×10^9^)±(8.26 ×10^8^) | 0.293^NS^ |
| Hemoglobin | 15.17±0.31 | 14.62±0.44 | 0.113^NS^ |
| Platelets | (7.96×10^11^)±(1.04 ×10^10^) | (7.79×10^11^)±(2.45 ×10^10^) | 0.315^NS^ |

Data expressed as Mean ±SD.^*^, ^**^ and NS indicate p < 0.05, p < 0.01 and not significant, respectively.
